# Photoinduced Charge Transfer and Trapping on Single Gold Metal Nanoparticles on TiO_2_

**DOI:** 10.1021/acsami.1c13662

**Published:** 2021-10-13

**Authors:** Monica Luna, Mariam Barawi, Sacha Gómez-Moñivas, Jaime Colchero, Micaela Rodríguez-Peña, Shanshan Yang, Xiao Zhao, Yi-Hsien Lu, Ravi Chintala, Patricia Reñones, Virginia Altoe, Lidia Martínez, Yves Huttel, Seiji Kawasaki, Alexander Weber-Bargioni, Victor A. de la Peña ÓShea, Peidong Yang, Paul D. Ashby, Miquel Salmeron

**Affiliations:** † IMN-Instituto de Micro y Nanotecnología (CNM-CSIC), 28760 Tres Cantos, Spain; ‡ Photoactivated Processes Unit, IMDEA-ENERGIA, 28935 Móstoles, Spain; § Departamento de Ingeniería Informática, Escuela Politécnica Superior, Universidad Autónoma de Madrid, Campus de Cantoblanco, 28049 Madrid, Spain; ∥ Departamento de Física, Universidad de Murcia, Campus de Espinardo, 30100 Murcia, Spain; ⊥ Molecular Foundry, Lawrence Berkeley National Laboratory, Berkeley, California 94720 United States; # Instituto de Ciencia de Materiales de Madrid (ICMM-CSIC), 28049 Madrid, Spain; ¶ Materials Sciences Division, Lawrence Berkeley National Laboratory, Berkeley, California 94720 United States; ∇ Materials Science and Engineering Department, University of California Berkeley, Berkeley, California 94720, United States; ○ Department of Chemistry, University of California, Berkeley, California 94720, United States

**Keywords:** charge transfer, photovoltage, Kelvin probe force microscopy, atomic force microscopy, TiO_2_, metal nanoparticles, photoelectrocatalysis

## Abstract

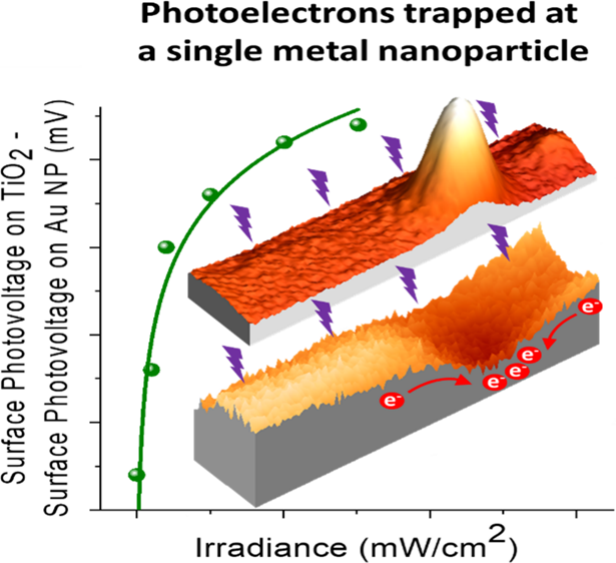

We present a study of the effect of gold nanoparticles (Au NPs) on TiO_2_ on charge generation and trapping during illumination with photons of energy larger than the substrate band gap. We used a novel characterization technique, photoassisted Kelvin probe force microscopy, to study the process at the single Au NP level. We found that the photoinduced electron transfer from TiO_2_ to the Au NP increases logarithmically with light intensity due to the combined contribution of electron–hole pair generation in the space charge region in the TiO_2_–air interface and in the metal–semiconductor junction. Our measurements on single particles provide direct evidence for electron trapping that hinders electron–hole recombination, a key factor in the enhancement of photo(electro)catalytic activity.

## Introduction

Many technologies of societal importance, such as CO_2_ conversion to fuels, hydrogen production by water splitting, and new materials with self-cleaning and antifogging properties, are based on heterogeneous photocatalysis.^1−3^ A limitation of these technologies is their low efficiency due to the high recombination rate of the photogenerated electron–hole pairs. Strategies to increase charge separation in the photocatalyst include decorating the surface with metal nanoparticles. The Schottky barrier at the metal–semiconductor interface and the associated electric field in the space charge region increases the efficiency of separation of electrons and holes and their diffusion.^4^ Under UV illumination, metal NPs in direct contact with the semiconductor can efficiently attract photogenerated electrons from the TiO_2_ conduction band, while holes are accumulated in the valence band of the oxide. This spatial separation of charge carriers results in an increased lifetime and, therefore, the catalytic efficiency. This strategy significantly increases the photocatalytic reduction efficiency of CO_2_, the selectivity toward methane synthesis,^5,6^ the photoproduction of hydrogen,^7^ and the photo-oxidative degradation of NO by increasing the efficiency by 700% relative to unmodified TiO_2_.^8^ In addition, studies reveal that interfacial sites at the TiO_2_–Au NPs activate adsorbed molecular species.^9,10^ However, the charge-transfer process at the TiO_2_–metal NP junction scale is poorly understood, and yet, it is key to enable the design of more efficient photoactive materials.

Another recent strategy to improve the catalytic performance is the use of plasmonic properties of Au nanoparticles. The enhancement of the local electric field on the metal nanoparticle increases the local transition probability of an instantaneous one-photon reaction, favoring the photochemical reactions on and near the surface of the metal.^11^ The enhanced near-field in metal NPs has been shown to boost the excitation of electron–hole pairs in TiO_2_ and therefore increase the efficiency of the photocatalysis.^12^ Nonetheless, it should be taken into account that the presence of metal nanoparticles could also result in an enhancement or quenching, depending on the distance between the metal and the TiO_2_.^13^

The Au/TiO_2_ interface has been the subject of theoretical and experimental investigation. Theoretically, metal–semiconductor contact theory^14^ and density functional theory^15^ are most frequently employed. On the experimental side, ultrahigh vacuum studies using scanning tunneling microscopy and atomic force microscopy (AFM) have been used for atomic-scale characterization of TiO_2_ sensitized with metal clusters^16,17^ and with organic dyes used in solar cells,^18^ which contributed to our current knowledge of charge transfer in model metal/TiO_2_(110) systems,^19^ including Pt^20^ and small Pt clusters.^21^ The effect of light exposure has been studied using photoluminescence,^22^ photoelectron spectroscopy, and surface photovoltage (SPV) techniques^4^ and used for the evaluation of photocatalytic activity.^23^ These experiments suggest that electrons are transferred from TiO_2_(110) to the Pt clusters.^20^ When the sample was afterward exposed to air and to N_2_ environments, no net charge transfer was detected,^24^ obviating the need for controlled experiments under ambient conditions. In addition, all these studies were performed over large areas, which average the effect of large numbers of NPs of different shapes and sizes and thus do not provide the critical microscopic insights needed to understand charge transfer between single NPs and the substrate.

Here, we focus on single Au NPs using Kelvin probe force microscopy (KPFM), which provides nanometer-scale resolution topographic and contact potential difference (CPD) maps. In addition, to overcome the averaging effect mentioned above, we focused a UV photon beam (*h*ν > 3 eV) onto the AFM tip while imaging. This operation mode, photoassisted Kelvin probe force microscopy (PA-KPFM), allowed us to map out the spatial topography and SPV structure on and around single Au NPs on TiO_2_ under illumination and their charge transfer and trapping properties in different environments. Our PA-KPFM results provide information on charge generation and transfer at the interface of individual Au NPs on TiO_2_. We then compared our results with those from space-averaging electrochemical techniques to reveal the fundamental processes that determine the enhancement in photocatalytic H_2_ production.

## Results and Discussion

### Photoexcited Carrier Generation on TiO_2_ in Gas-Phase Environments

**Figure 1:**
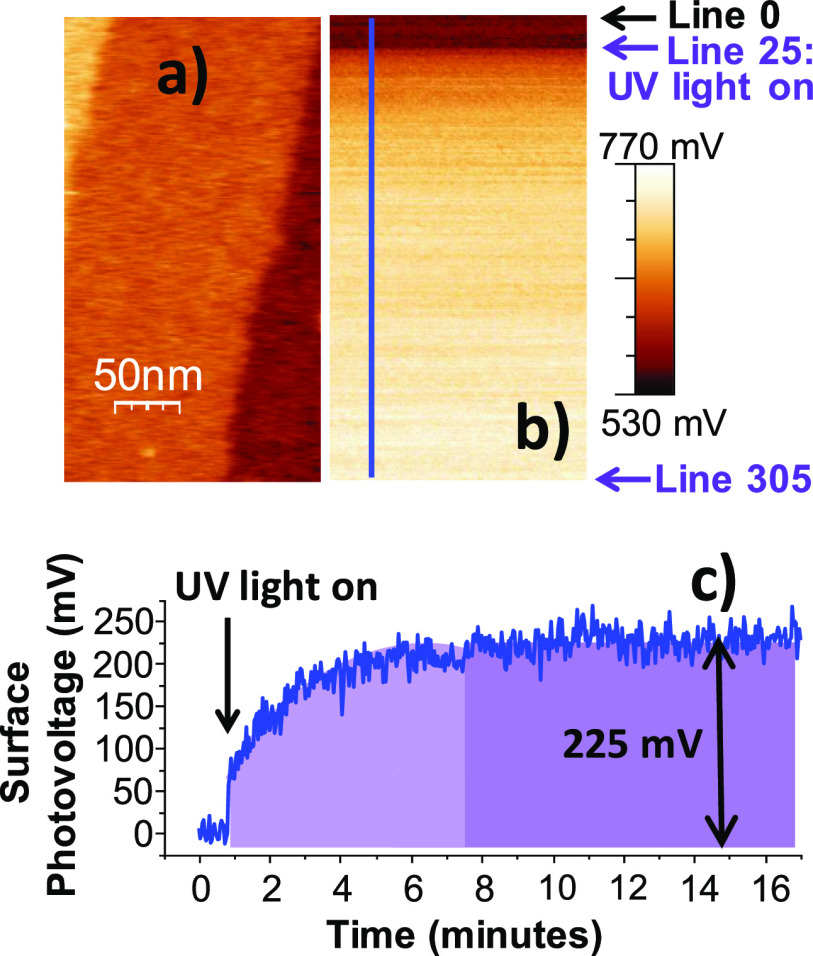
(a) PA-KPFM topography (the color scale span: 1.83 nm) and (b) *V*_CPD_ images of bare TiO_2_(110) acquired simultaneously. During the first minute, there was no UV illumination (lines 0 to 25). In the following lines, the UV illumination intensity was 0.40 mW/cm^2^. (c) Surface photovoltage (SPV = *V*_CPD-light_ – *V*_CPD-dark_) vs time (profile along the blue line in b). A total increase of 225 mV is measured.

We studied the bare TiO_2_(110) surface during illumination with supra-bandgap energy light (365 nm wavelength, corresponding to 3.4 eV) by scanning an AFM PtIr_5_ tip in noncontact mode while simultaneously acquiring topographic (Figure 1a) and CPD (*V*_CPD_) images (Figure 1b). During the first minute (top 25 lines of the image), the illumination was off, and then it was turned on for the remainder of the image. After turning the light on, a sudden increase of the *V*_CPD_ was observed by the change in contrast after line 25. The total light exposure time from line 25 to line 305 at the bottom of the image was 16 min. Figure 1c shows a *V*_CPD_ profile extracted from Figure 1b after subtracting the profile obtained in the dark. The resulting difference corresponds to the SPV.^25^ In the dark, the *V*_CPD_ was around +532 mV, with the positive value reflecting the higher work function of the metallic PtIr_5_ tip relative to the oxide substrate, as illustrated in the Supporting Information (Figure S1).

After the initial rapid increase, the SPV continued to increase slowly (light-purple region in Figure 1c) and saturated after about 5 min. The increase in SPV is due to the accumulation of positive carriers (holes) on the surface of TiO_2_ as expected from its n-type nature, which produces an electric field in the surface space charge region that drives the photogenerated holes to the surface. Although e–h pairs are generated throughout the penetration depth of the photons, which is on the order of micrometers, most of them recombine and only a fraction of those generated within the depletion region reach the surface. These charge carriers can be temporarily captured on traps created by defects. The photoexcited holes accumulated at the surface counter-balance the built-in surface potential, causing the bands to flatten partially. The flattening or downward bending of the TiO_2_ bands upon illumination manifests in a decrease in the sample work function so that the *V*_CPD_ is more positive (larger) under illumination, as shown in the Supporting Information (Figure S2). Electron excitation from defect states can also occur under UV-light illumination.^26^

When the light was turned off, the time to recover the original SPV value was on the order of days due to the long lifetime of trapped holes on the surface of the TiO_2_ in the inert N_2_ atmosphere of our chamber. However, in the presence of hole-scavenger species, the recovery time decreased to a few minutes. We demonstrated this by introducing methanol, an active reducing agent, into the KPFM chamber with the N_2_ (Figure S3).

### Photoexcited Carriers on TiO_2_ in Electrolyte Environments

**Figure 2:**
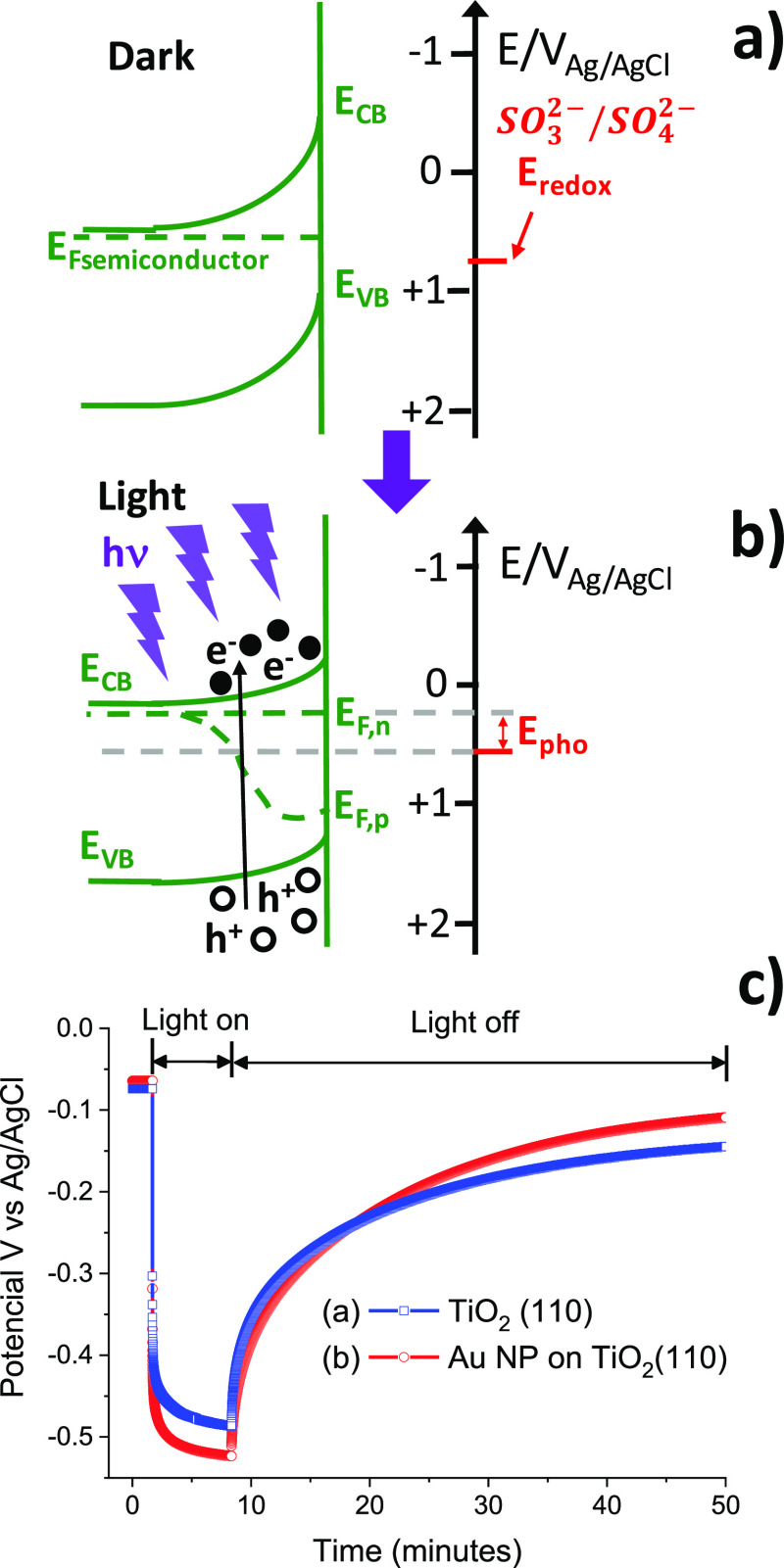
PV in an electrochemical cell containing 0.5 M Na_2_SO_3_ aqueous solution at pH 9. (a) When the TiO_2_ sample is submerged in the electrolyte, the Fermi level is pinned at the redox potential level of the electrolyte (in this case, SO_3_^2–^*E*_redox_), which is located in the TiO_2_ band gap. The bands shift to more positive potentials for an n-type semiconductor, with a resultant upward bending. (b) Under UV light, photogenerated minority carriers (holes) move to the surface where they are compensated by negatively charged species from the electrolyte, decreasing the band bending and causing the potential to move to more negative values (vs Ag/AgCl reference electrode). (c) Sample potential measured under illumination by band gap UV light on TiO_2_ with and without Au NP as a function of time.

In parallel experiments, photovoltage (PV) measurements were carried out in a photoelectrocatalytic cell filled with a 0.5 M Na_2_SO_3_ aqueous solution at pH 9. The experiments were carried out both with bare TiO_2_ and with ligand-free Au NPs covering the TiO_2_(110). Electron exchange across the TiO_2_–electrolyte junction equilibrates the sample Fermi level with the redox potential of the SO_3_^2–^/SO_4_^2–^ pair, which is located in the band gap of TiO_2_. As a result, the bands of the oxide shift upward at the interface, as illustrated in Figure 2a. When the light was turned on, a fast negative change in the sample voltage versus Ag/AgCl, followed by a slower decrease reaching a quasi-steady state, was observed. The photogenerated carriers (holes) move to the surface of the semiconductor, where they are compensated by negatively charged species from the electrolyte, leading to a decrease in the band bending. This is in line with the SPV measurements by PA-KPFM in the gas environment described earlier, where positive values indicate the presence of more holes at the surface.

A noticeable difference in recovery time to the original PV value after turning off the illumination is observed between the PA-KPFM experiments performed under a gas atmosphere (Figure 1) and the photoelectrochemistry results (Figure 2). In the electrolyte, the decay time is approximately a couple of hours, while in a N_2_ atmosphere, it takes several days. The difference is due to the presence of SO_3_^2–^ charge-scavenging ions in the solution, which react with the created holes and transfer the charge to the solution, similar to the effect of methanol in the gas-phase experiments described above.

### Effect of Au NPs on Photoexcited Carriers in TiO_2_ in Gas-Phase Environments

**Figure 3:**
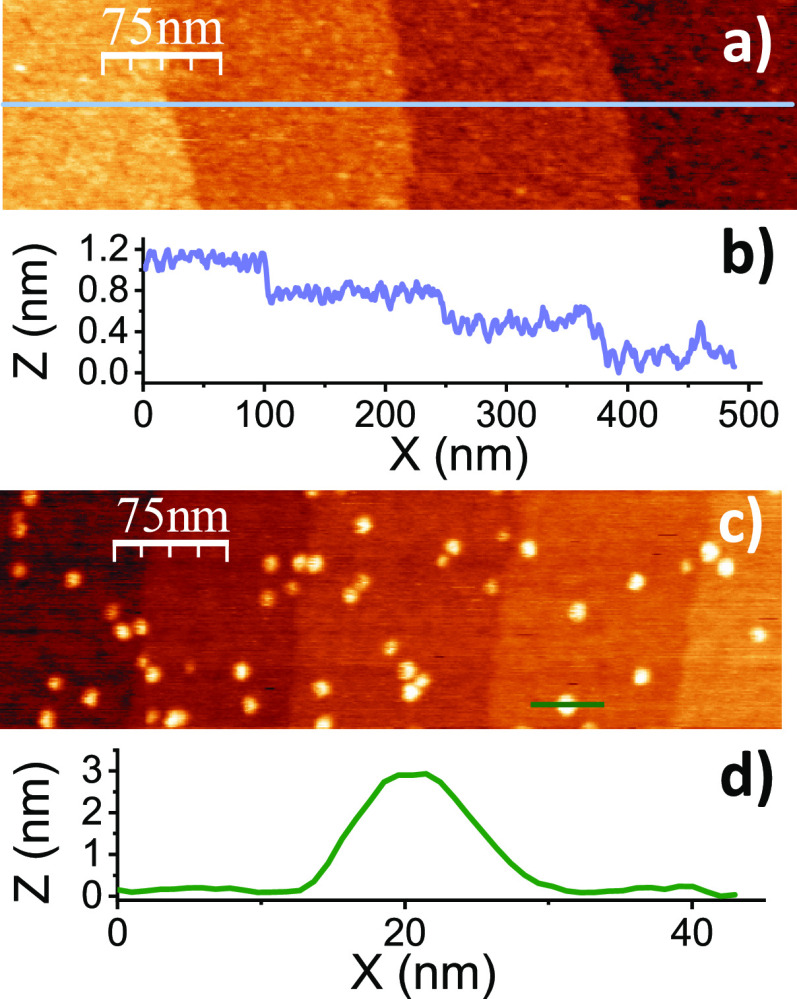
AFM topographic images of TiO_2_(110). (a) Without and (c) with deposited gold nanoparticles. (b,d) Height profiles along the lines in (a,c), respectively.

After studying the charge generation and photovoltaic behavior of bare TiO_2_, we focused on the effect of the Au NPs on the photovoltaic and chemical properties of TiO_2_. Figure 3 shows noncontact amplitude-modulation topographic images of TiO_2_(110) before (Figure 3a,b) and after decoration with ligand-free Au NPs (Figure 3c,d). The surface coverage is 0.42%. The height profiles along the lines in the figure indicate a step height of 0.33 nm on the TiO_2_ surface, and a Au NP height of 3.0 ± 0.6 nm, which corresponds to the NP diameter. A histogram of the Au NP size for this sample is shown in Figure S4 in the Supporting Information.

**Figure 4:**
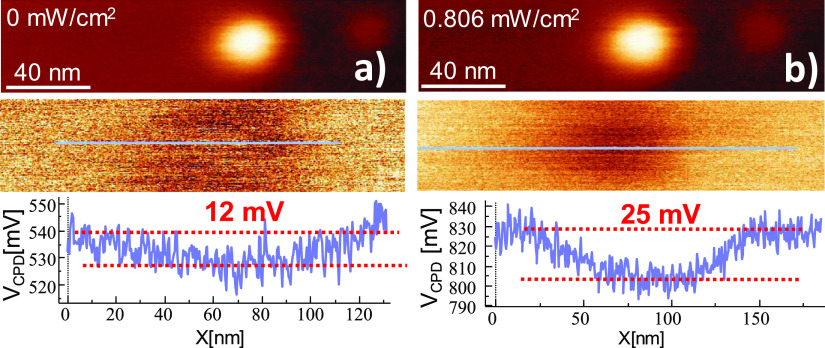
Topography (top) and *V*_CPD_ (bottom) images of a 3 nm diameter Au NP on bare TiO_2_(110) (a) in the dark and (b) under UV illumination. *V*_CPD_ profiles are shown at the bottom. Color scale in topography: (a) 3.6 nm and (b) 4 nm. A CPD image of a larger sample area can be found in the Supporting Information (Figure S10).

**Figure 5:**
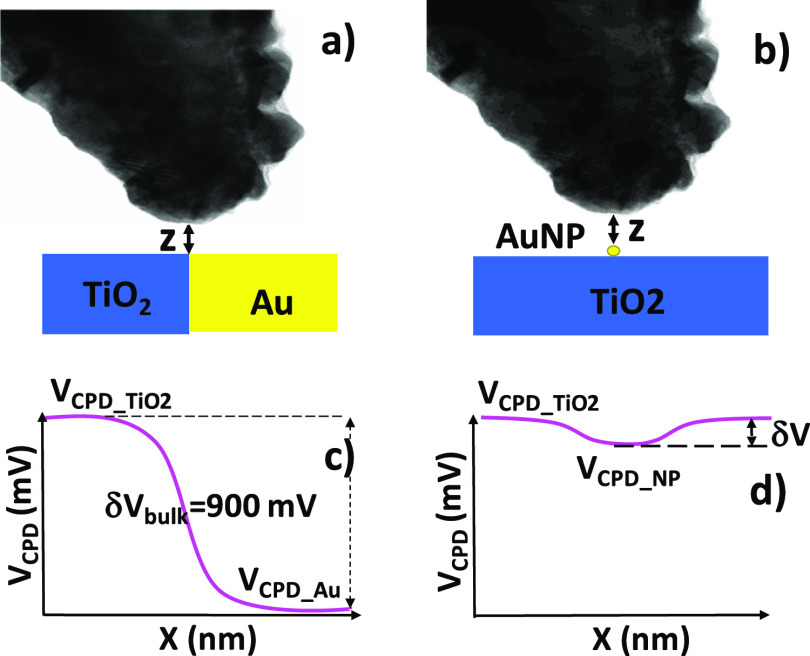
Calculated averaging effects of the CPD measured by the tip on Au NP versus bulk Au (details in the Supporting Information, sections 7 and 8). (a) Scheme where KPFM is performed on a sample consisting of bulk TiO_2_ and Au; (b) same for a Au NP on the TiO_2_ surface. The theoretical modeling predicts a significant reduction on the CPD (δ*V*) in (b) due to the small size of the Au NP with respect to the tip.

Using PA-KPFM, we then measured the CPD and the effect of light intensity around individual Au NPs in the dark and under UV illumination. As shown in Figure 4a, in the dark, the CPD is 12 mV lower (more negative) on top of the 3 nm Au NP than the surrounding TiO_2_. The width of the CPD profiles is about 50 nm, which is about the diameter of the tip (see tip apex TEM image in Figure S5). The difference in CPD measured on top of the Au NP compared to the TiO_2_ substrate reflects the higher work function of Au (*W*_Au_ = 5.1 eV) compared to that of TiO_2_(110) (*W*_TiO2_ = 4.2 eV) that leads to a transfer of negative charge from TiO_2_ to the Au NP, which creates a Schottky barrier at the Au/TiO_2_ interface with a calculated depletion length^14^ of about 12 nm for NPs of 3 nm diameter. Details of this calculation can be found in the Supporting Information (Figure S6). Although the small size of the Au NP is known to lead to a smaller work function than that of bulk Au,^27^ the small measured value of 12 mV compared to the difference in the work functions of bulk Au and TiO_2_ (Figure 5) is mostly due to averaging effects due to the large tip apex radius. A theoretical modeling (Supporting Information sections 7 and 8) shows that a significant reduction in the measured surface potential difference between TiO_2_ and the Au NP (Figure 5b) of 87% is indeed expected with respect to bulk Au due to averaging (Figure 5). The model also indicates that the field produced by the charge in the NP is reduced by that emanating from the image charge of opposite sign in the supporting TiO_2_.

**Figure 6:**
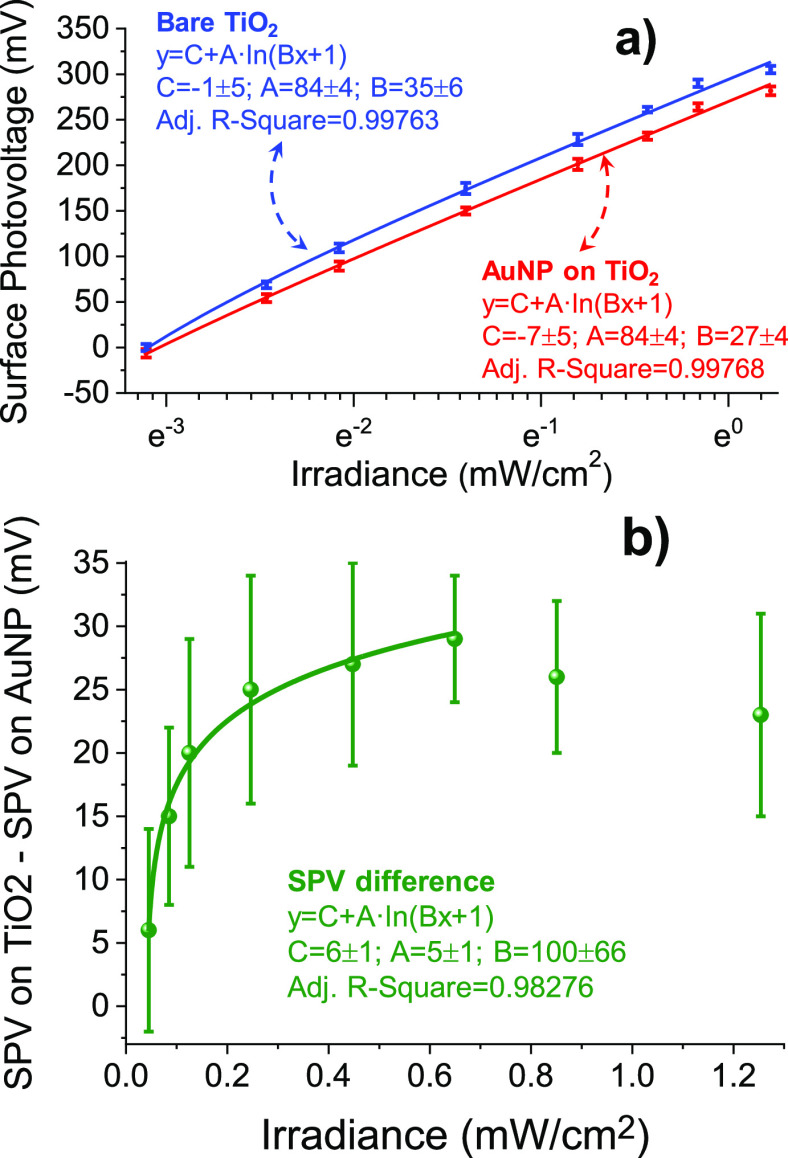
(a) Surface PV vs irradiance with 365 nm wavelength light, with fits to the logarithmic functions for bare TiO_2_ (blue) and for the Au NP on the TiO_2_ (red). (b) Difference between SPV on bare TiO_2_ and SPV on the Au NP as a function of irradiance. The data in (a) is plotted with a logarithmic *X*-axis, while the data in (b) has a linear axis. The differences shown in (b) underestimate the real difference because the SPV on top of the Au NP averages with the value of the SPV on the surrounding area, as explained in Figure S6 of the Supporting Information.

Under illumination, the CPD on top of the Au NP is lower by 25 mV relative to the surrounding TiO_2_, larger than the value in the dark by factor 2. To understand this result, we should consider that there are two space charge regions in the TiO_2_, one in the vicinity of the Au NP/TiO_2_ (metal–semiconductor space charge region) and the other at the interface between the TiO_2_ surface and the gas away from the NP (Supporting Information, Figure S6a). Therefore, with UV irradiation, the photogenerated carriers will diffuse to the two interfaces in different amounts. Our results indicate that the Au NPs are more negative during illumination than the surface areas of TiO_2_ farther away from them. The resulting differential charging was measured by plotting the difference in *V*_CPD_ of Au versus that of TiO_2_ as a function of irradiance (Figure 6a), with each point representing the mean value of 144 data points. Figure S11 (Supporting Information) shows simultaneous topography and CPD images at each value of the irradiance. As the light power increases, the SPV increases, reaching a value of 300 mV at an irradiance of 1.25 mW/cm^2^. The logarithmic dependence on light intensity is typical for charge separation at the built-in potential (before saturation) in the space charge region,^28^ which decreases the band bending due to accumulation of positive carriers at the surface. In the fit, , η and B are proportionality constants that depend on the state of the surface and on the photoelectromotive force (emf) mechanism, and I is the illumination intensity. From the fit to the data, we obtain η ≈ 3  = 25.7 mV at *T* = 300 K. Values above 2 are characteristic of significant trapping.^28,29^ In Figure 6b, we plot the SPV difference between the values on top of Au and on the bare TiO_2_, which can be fitted by a logarithmic function with a coefficient of 6 ± 1, indicating that the Au NPs are efficient at trapping negative charge as the irradiance increases. The Au NPs are more efficient than suggested by the measurement because the SPV measured on top of the Au NPs is an average between that on the Au and that of the surrounding area within a radius of the order of the tip diameter. Although the maximum difference measured depends on parameters such as tip size, the logarithmic trend (Figure 6a,b) was found in all the experiments. Figure 6b also shows that after increasing from 0 to 0.6 mW/cm^2^, the SPV diminishes at high intensities. This can be attributed to the Dember effect,^30^ where high irradiance gives rise to differences in photogeneration rates along the photon penetration depth and space charge region. This, coupled with the different electron and hole diffusivities decreases the value of the SPV due to the higher hole concentration near the TiO_2_ surface.^1,31^

Effects on the SPV from defects and impurities of the TiO_2_ surface are not expected to play a significant role here. For example, surface states from O-vacancies should not be present in appreciable amounts due to the annealing treatment performed under a flux of clean air. Finally, the presence of organic contamination, such as small-chain carboxylic acids, is not expected to affect photocharge separation because the organic compounds do not absorb UV light efficiently. Moreover, organic compounds will be removed from the surface under UV illumination, which oxidizes and eliminates them from the surface. To demonstrate the photo-degradation of carbonaceous species, we performed experiments using highly surface-sensitive sum frequency generation spectroscopy (Supporting Information, section 11).

It is interesting to note that gold NPs absorb light from the visible region of the spectra (530 nm), attributed to plasmon resonance wavelength. However, in Au NPs on TiO_2_ systems,^5^ the UV irradiation does not show a significant effect in the SPR of Au NPs. This is mainly because the main portion of the UV irradiation is absorbed by TiO_2_. The fraction that can be absorbed by the Au NPs generates hot electrons^32^ that quickly recombine, leading to a negligible effect in the charge dynamic processes. In some cases, this recombination could lead to a local increase of the temperature in the Au NPs (hot spot), which could have some effect in the catalytic reaction.

### Effect of Au NPs on Photoexcited Carriers on TiO_2_ in Electrolyte Environments

**Figure 7:**
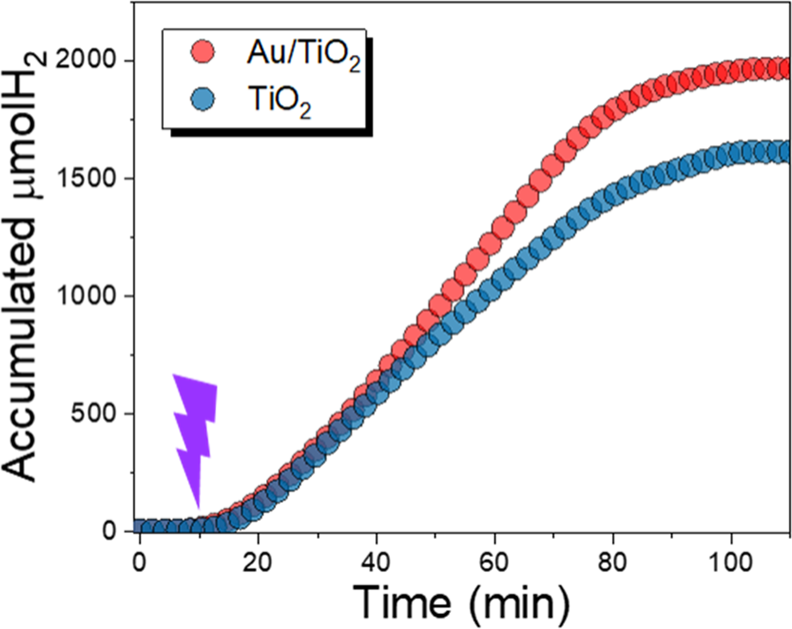
Accumulated H_2_ production for TiO_2_ and Au NPs on TiO_2_ under UV irradiation at 0.7 V (vs Ag/AgCl) during 1 h. The light was turned on after a stabilization period of 10 min.

As shown in Figure 2, in an electrochemical environment, the samples with Au NPs also show a photovoltage difference (vs Ag/AgCl reference) relative to bare TiO_2_, although in this case, the value represents an average over the whole surface. In the electrochemical environment, a large change in photovoltage is also observed when Au NPs are present, also following a logarithmic behavior versus irradiance (Supporting Information, Figure S15c). The PV on the sample with Au NPs reaches a larger minimum, implying that more trapped charges are available on this surface than on the surface without Au NPs. Moreover, electrochemical impedance spectroscopy (EIS) confirms that the presence of Au NPs improves the conductivity of electrons through the TiO_2_ to the solution and determines the flat band potential (*V*_FB_) and Fermi level position of both samples (Supporting Information, Figures S16 and S17). These results demonstrate the presence of a change in band positions when Au NPs are supported in the surface (Figure S18b). Photoelectrocatalytic experiments (in aqueous solution) were performed to evaluate the benefits of Au NPs on catalyst performance. Under UV illumination, we found that the accumulated hydrogen production increases during illumination and reaches a value of 1950 μmol H_2_ at the end of the reaction (Figure 7), which is more than 30% higher than in bare TiO_2_. Photocurrent measurements exhibited a similar behavior, showing a current decrease, in the case of TiO_2,_ more accentuated even at the first stage of the reaction (Figure S19). This behavior indicates that in both cases, the main active sites for photoelectrocatalytic reactions are located on the TiO_2_ surface. Therefore, Au nanoparticles act as capacitive junctions that improve the photoinduced current, leading to a spatial separation of charge carriers and therefore a decrease in the recombination rate in e^–^–h^+^ pairs, enhancing the catalyst stability.

## Conclusions

In this work, we elucidate the effect that the Au NP decorated on TiO_2_ has on the photogeneration, charge transfer, and trapping. By examining the effect of illumination on single Au NPs we were able to relate nanoscale observations with macroscopic results from TiO_2_/AuNP–electrolyte interfaces under different conditions. By means of PA-KPFM, we have been able to achieve high-resolution SPV imaging of single Au NPs of 3 nm diameter simultaneously with the topographic image. This allowed us to compare the differential charge transfer to the Au NP and to the TiO_2_ surface when the system was exposed to light of band gap energy and to measure its dependence on light irradiance.

Our results indicate that a substantial number of electrons are transferred from the TiO_2_ to the metal nanoparticle, which increases with light intensity in a logarithmic fashion. The logarithmic increase was explained by the interplay between the bare TiO_2_ surface space charge region and the metal–semiconductor space charge region and their dependence on irradiation intensity. Irradiation induces a reduction of the TiO_2_ work function due to band flattening, which in turn produces a larger bending of the Schottky barrier at the interface between the titania TiO_2_ surface and the metal nanoparticle, thus promoting electron transfer to the metal nanoparticle. Therefore, because of the fact that the reduction of the surface space charge region with light power has a logarithmic rate, a logarithmic behavior is also expected for the electron transfer versus irradiance, as our results indicate.

Finally, we have shown that the observed electrochemical behavior of TiO_2_ photoelectrodes containing large amounts of Au NPs is in line with the conclusions obtained by PA-KPFM. This parallel study by PA-KPFM and electrochemical experiments provides a deeper understanding of the charge transfer to the metal NP and to the TiO_2_ and allows us to correlate the photoinduced charge generation and transfer in Au/TiO_2_ interfaces with the enhancement in photocatalytic H_2_ production.

## Experimental Section

### TiO_2_ Substrate Preparation

The TiO_2_(110) surface (Nb doped by 0.05% of the total weight, Shinkosha, CO. LTD.) was annealed in a preheated furnace at 900 °C for 1 h under a continuous flow of air.

### Au NP Fabrication and Deposition

The Au NPs were prepared by means of a magnetron-sputtering–multiple-ion-cluster source^33,34^ and deposited on Nb-doped TiO_2_(110) crystals that were previously annealed in air. This gas-phase synthesis produced crystalline Au NPs with a convex regular icosahedral structure.^35^ Details of the sample preparation procedure are presented in the Supporting Information (section 4).

### PA-KPFM Implementation and Calibration

A fiber-coupled LED (M365FP1, Thorlabs Inc.) was used for illuminating the sample (wavelength 365 nm). The light beam was introduced in the optical path of a Cypher ES (Asylum Research, Oxford Instruments) AFM head through a dichroic hot mirror. We checked the consistency and stability of the PtIr tip work function by performing AP-KPFM measurements on graphite because the CPD is insensitive to light exposure when the metal coating is intact. The illumination calibration was performed with the silicon photodiode (UV extended) S120VC from Thorlabs. More details can be found in the Supporting Information (section 13).

### PA-KPFM Experiments

PA-KPFM was implemented by operating in noncontact amplitude modulation mode (AM-AFM) with phase-locked loop (PLL) detection and force gradient feedback.^36^ In this technique, two different types of modulations were applied to the cantilever: (1) a mechanical excitation at its resonance frequency and (2) an electrical excitation at lower frequency. As the tip scanned the sample, three types of simultaneous feedback were performed: (1) topography feedback to keep the mechanical oscillation amplitude constant by regulating the tip–sample distance (AM-AFM);^37,38^ (2) a phase-locked loop (PLL) nullifies the phase difference between the driving signal and the cantilever mechanical oscillation, keeping the oscillation at resonance by controlling the excitation frequency; (3) a bias voltage is applied to the tip in order to nullify the electrostatic force or force gradient.^39^ Hence, the CPD between the tip and sample is obtained at each pixel, as described in published literature.^40,41^ We used a Cypher ES (Asylum Research) AFM and ATEC-EFM (Nanosensors) cantilevers, with a resonance frequency of 85 kHz and a force constant of 2.8 N/m. For the applied electrical excitation, the chosen amplitude was 2 V (rms) at a frequency of 7 kHz.

A key point of our technique is to achieve stable noncontact operation. Topographic noncontact feedback relies on the attractive force between the tip and sample that decreases the oscillating amplitude of the cantilever as the tip approaches the surface, making possible noncontact imaging of the surface and simultaneous acquisition of topography, frequency, and CPD data in a single pass.^40−42^ It should be noted that the resolution in the AFM images is determined by the diameter of the tip apex, which is around 40 nm in our experiments, as shown in the Supporting Information (Figure S5). Data processing was done using the free software WSxM (version 5.0).^43^

### Photo(electro)chemical Experiments

The studies with single Au NPs on TiO_2_ were complemented by spatially averaged studies (i.e., with many NPs on the TiO_2_) using electro- and photoelectrochemical measurements in a three-electrode cell with a quartz window in an aqueous solution of 0.5 M Na_2_SO_3_ at pH 9. Both Au NPs/TiO_2_ and bare TiO_2_ samples (5 × 5 mm^2^) were used as working electrodes. The counter electrode was a platinum wire, and a Ag/AgCl wire was used as the reference electrode. The currents, in the dark and under illumination and the voltage, were measured with a potentiostat–galvanostat PGSTAT302N equipped with an integrated impedance module FRAII. A modulation amplitude of 10 mV was used in the frequency range from 1 Hz to 10,000 Hz in the EIS measurements. The experiments were conducted under an argon flow of 50 sccm through the top of the cell. A UV LED lamp (80 mW/cm^2^) was used as the light source. To measure the reaction products, the cell was connected to a gas chromatograph (Agilent micro-GC 490) equipped with a MS5A column at a temperature of 60 °C and a TDC detector.

## Supporting Information Available

Kelvin Probe Force Microscopy (KPFM) operation principle; Surface Photovoltage (SPV) under illumination exposure in Kelvin Probe Force Microscopy; SPV decrease in the presence of a hole scavenger; Au NPs fabrication and deposition on the TiO_2_(110) surface. Au NPs size analysis; TEM image of conductive tip apex; Length of the Schottky junction depleted region for finite and small Au nanoparticle; Theoretical calculation of the electrostatic interaction forces between tip and surface; Theoretical model for calculating electric charge inside the nanoparticle; Au NP on TiO_2_ CPD image of large areas; Topography, VCPD and Resonance Frequency images of an Au NP on TiO_2_(110) by means of KPFM; Evaluation of carbonaceous molecular species on TiO_2_(110) by means of sum frequency generation spectroscopy; Photoelectrochemical characterization; AFM modification for light implementation and light calibration (PDF)
