# Supplementary material for: Photoinduced Charge Transfer and Trapping on Single Gold Metal Nanoparticles on TiO2
Source: ACS Appl Mater Interfaces. 2021 Oct 13;13(42):50531–8. doi: 10.1021/acsami.1c13662 (PMC8554764; doi:10.1021/acsami.1c13662)
Supplement: Supplementary file 1 — am1c13662_si_001.pdf [file am1c13662_si_001.pdf]

Supporting Information

Photoinduced Charge Transfer and Trapping on Single Gold Metal Nanoparticles on TiO_2

Monica Luna[§]*, *Mariam Barawi*[├], *Sacha Gómez-Moñivas*[♦], *Jaime Colchero*[☼], *Micaela Rodríguez-Peña*[§], *Shanshan Yang*[⸸], *Xiao Zhao*[⸸], *Yi-Hsien Lu*[‡⸸], *Ravi Chintala*[‡], *Patricia Reñones*[├], *Virginia Altoe*[‡], *Lidia Martínez*[†], *Yves Huttel*[†], *Seiji Kawasaki*[‡⸸], *Alexander Weber-Bargioni*[‡], *Victor A. de la Peña O´Shea*[├], *Peidong Yang*[⸸#], *Paul D. Ashby*[‡]*, *Miquel Salmeron*[⸸⊥]*

[§]IMN-Instituto de Micro y Nanotecnología (CNM-CSIC), 28760 Tres Cantos, Spain

[├]Photoactivated Processes Unit, IMDEA-ENERGIA, 28935, Móstoles, Spain

[♦]Departamento de Ingeniería Informática, Escuela Politécnica Superior, Universidad Autónoma de Madrid, Campus de Cantoblanco, Madrid 28049, Spain

[☼]Departamento de Física, Universidad de Murcia, Campus de Espinardo, Murcia, Spain.

[‡]Molecular Foundry, Lawrence Berkeley National Laboratory, California, 94720 United States

[†]Instituto de Ciencia de Materiales de Madrid (ICMM-CSIC), 28049 Madrid, Spain

[⸸]Materials Sciences Division, Lawrence Berkeley National Laboratory, California, 94720 United States

[⊥]Materials Science and Engineering Dept. University of California Berkeley, California 94720, United States

[#]Department of Chemistry, University of California, Berkeley, California 94720, United States

Corresponding Authors
*monica.luna@csic.es

*pdashby@lbl.gov

*mbsalmeron@lbl.gov

Supporting Information

Index:

1. **Kelvin Probe Force Microscopy (KPFM) operation principle.**
2. **Surface Photovoltage (SPV) under illumination exposure in Kelvin Probe Force Microscopy.**
3. **SPV decrease in the presence of a hole scavenger.**
4. **Au NPs fabrication and deposition on the TiO_2(110) surface. Au NPs size analysis.**
5. **TEM image of conductive tip apex.**
6. **Length of the Schottky junction depleted region for finite and small Au nanoparticle.**
7. **Theoretical calculation of the electrostatic interaction forces between tip and surface.**
8. **Theoretical model for calculating electric charge inside the nanoparticle.**
9. **Au NP on TiO_2 CPD image of large areas.**
10. **Topography, V_{CPD} and Resonance Frequency images of an Au NP on TiO_2(110) by means of KPFM.**
11. **Evaluation of carbonaceous molecular species on TiO_2 (110) by means of sum frequency generation spectroscopy.**
12. **Photoelectrochemical characterization.**
13. **AFM modification for light implementation and light calibration.**
14. **Bibliography.**

1. Kelvin Probe Force Microscopy (KPFM) operation principle.

KPFM is sensitive to local variations in the work function of materials. The work function depends on the specific material, adsorption layers (e.g., water), oxide layer thickness, dopant concentration, electrostatic charge, surface dipole moments and temperature.[1] The operation principle is described in Figure S1.

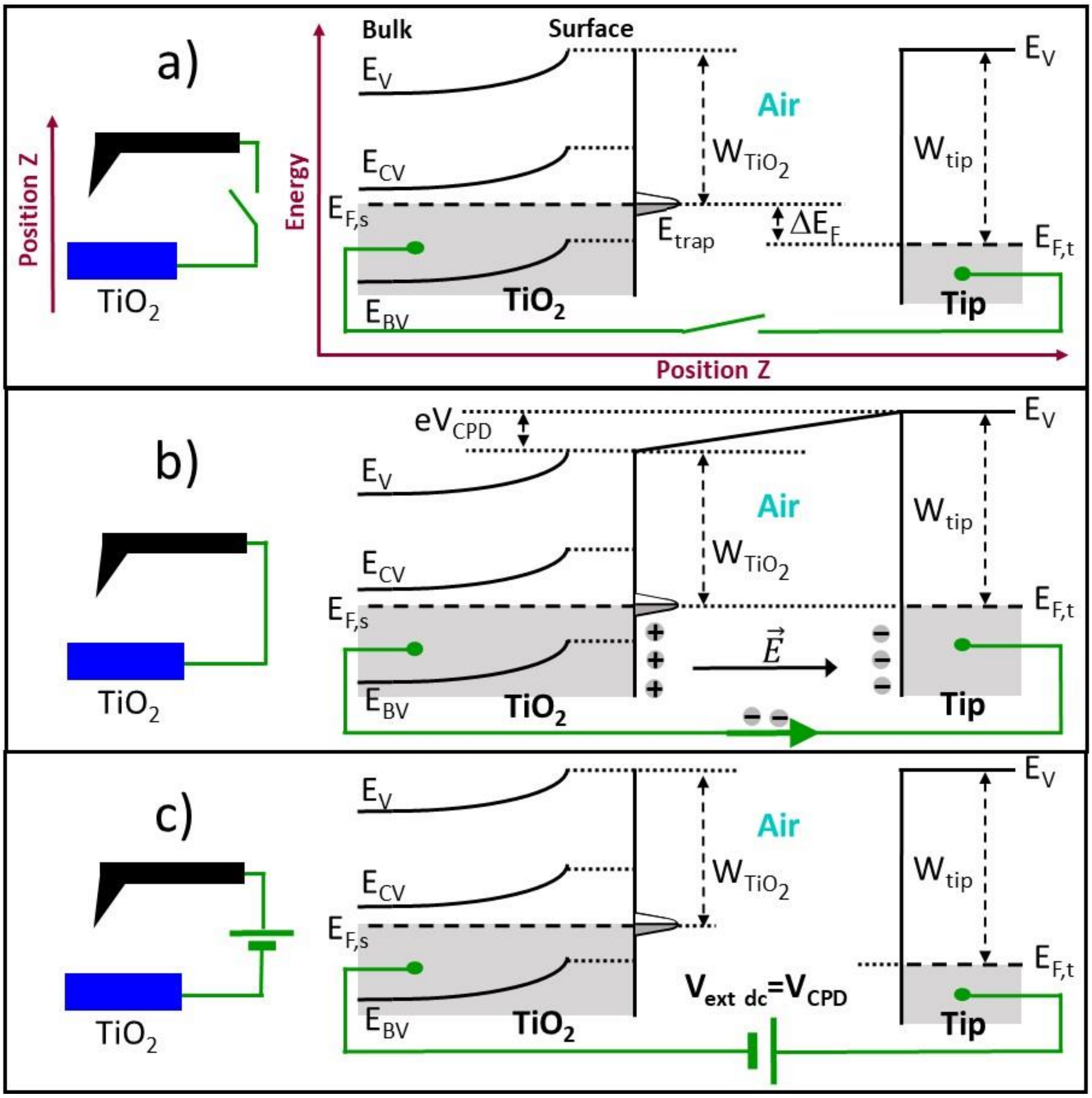

Figure S1. Kelvin Probe Force Microscopy operation principle. Semiconductor surface (bare TiO_2) and a metallic tip (PtIr5) when they are electrically disconnected (a), electrically connected (b) and under KPFM operation (c). In this particular case, the semiconductor work function (W) is smaller than the metal one: $W_{TiO2} = 4.2$ eV and $W_{tip} = 5.1$ eV (a). Therefore, when tip and sample are electrically contacted, electrons flow from the semiconductor to the tip (b) until both Fermi Levels are aligned, generating a contact potential difference (V_{CPD}) and an electric field (E)

between tip and surface. Under the KPFM operation (c), the electric field is nullified by applying and external voltage ($V_{ext\ dc}$) to the tip. In this case: $W_{TiO2} < W_{tip}$, therefore the tip acquires a positive voltage. With the values of the V_{ext} obtained at each point of the surface sample, the surface voltage is mapped.

2. Surface Photovoltage (SPV) under illumination exposure in Kelvin Probe Force Microscopy

The increase in SPV under illumination exposure can be explained by taking into account the upwards band bending in the semiconductor surface space charge region (Su-SCR) and as a consequence, the promotion of holes to the surface.

The depletion region depth[2] can be estimated from the Debye length, (*Ld*), and is about 3.5 nm for the sample under study:

$$L_d = \sqrt{\frac{\varepsilon_b \varepsilon_0 k_B T}{e^2(n_b+n_p)}} = 3.5 \text{ nm}$$

Since $n_b = 1.4.\ 10^{25}\ m^{-3}$, $n_p \approx 0$, *e* is the electron charge, $k_B T = 4.11\times10^{-21}$ J at room temperature, $\varepsilon_0 = 8.85\ 10^{-12}\ F{\cdot}m^{-1}$ and $\varepsilon_b = 120$. When the sample is illuminated with photons of energy above the semiconductor band-gap, electron-hole pairs are generated: electrons are excited from O_{2p} states to conduction band (O2p→ Ti3d), leaving behind holes. Most photo excited charge carriers recombine and release energy. A small fraction of the charge carriers ($e^- - h^+$) which are generated within the band bending region are able to diffuse before recombination happens. In n-type semiconductors holes travel towards the surface and electrons away from it, due to that interfacial electric potential gradient. Charge carriers at the surface can be temporarily captured by defect active sites (surface traps, shallow or deep traps) acting as trapping sites. Therefore, the increase of SPV corresponds to a decrease in the negative surface charge due to an increase in the number of photo-holes arriving at the surface per time unit. This partially counterbalances the built-in surface potential and therefore, the bands partially flatten.

Photo-Assisted Kelvin Probe Force Microscopy (PA-KPFM) was used to map out topography and surface photovoltage, which is obtained through the difference in contact potential voltage (V_{CPD}) under illumination and under dark conditions. With this technique we studied the changes in the carrier diffusion and trapping of isolated single 3 nm diameter Au nanoparticles supported

on TiO_2 under different environments. Figure S2 shows the energy band diagrams for tip and sample in darkness (thick black) and under illumination (thin purple). The metallic tip work function is not altered by light exposure. However, with irradiation, at the surface, all three bands lower their energy value by *e.SPV*:

$$E_{Vdarck} = e \cdot SPV + E_{Vlight},$$

$$E_{CBdark} = e \cdot SPV + E_{CBlight}$$

$$E_{VBdarck} = e \cdot SPV + E_{VBlight}$$

The flattening of the partial band bending upon illumination implies a decrease in the metal oxide work function (a). When tip and sample are electrically disconnected (a) their Fermi levels are not aligned. When connected (b), the alignment is achieved by the flow of electrons from the TiO_2 to the tip, creating an electrical field. The V_{CPD} is larger under illumination. Therefore, the larger the band flattening, the higher the V_{CPD} (positive) increase and, thus, the higher (positive) SPV. When operating in KPFM, an external potential is applied to the tip equal to the V_{CPD}, and thus removing the electrical field (E) (Figure S2).

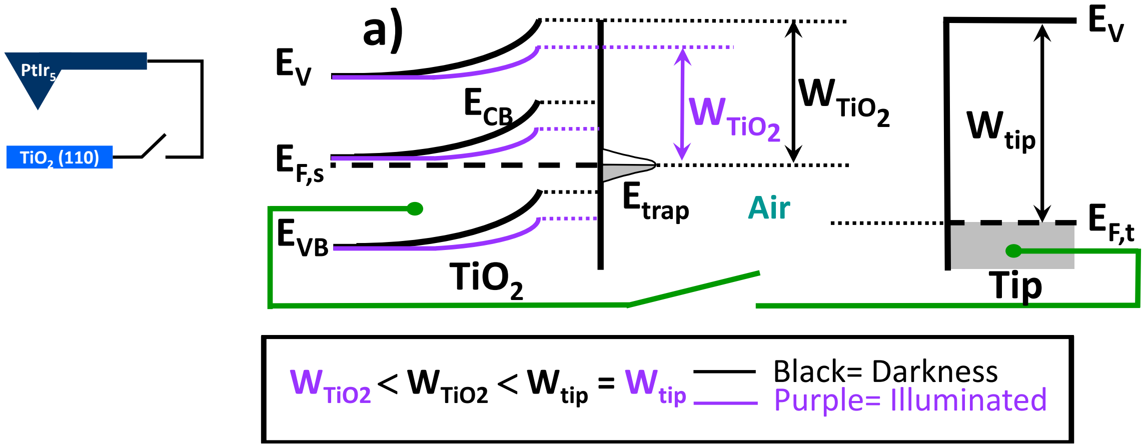

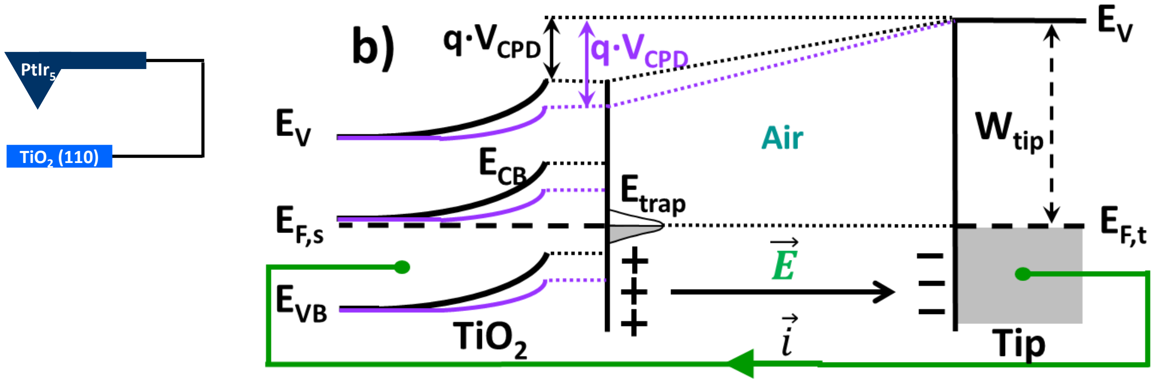

Figure S2. Band diagrams for tip (PtIr5) and sample (TiO_2, n-type) in the dark (black) and under UV light (purple) when the tip and sample are electrically disconnected (a) and connected (b). The light exposure causes the bands to partially flatten. At the surface: E_{CB}-E_F (dark) > E_{CB}+E_F (light). Accordingly, the V_{CPD} is larger under illumination and therefore the SPV value will be larger. SPV = V_{CDP} (light)-V_{CDP} (dark).

3. Surface Photovoltage (SPV) decreases in the presence of a hole scavenger.

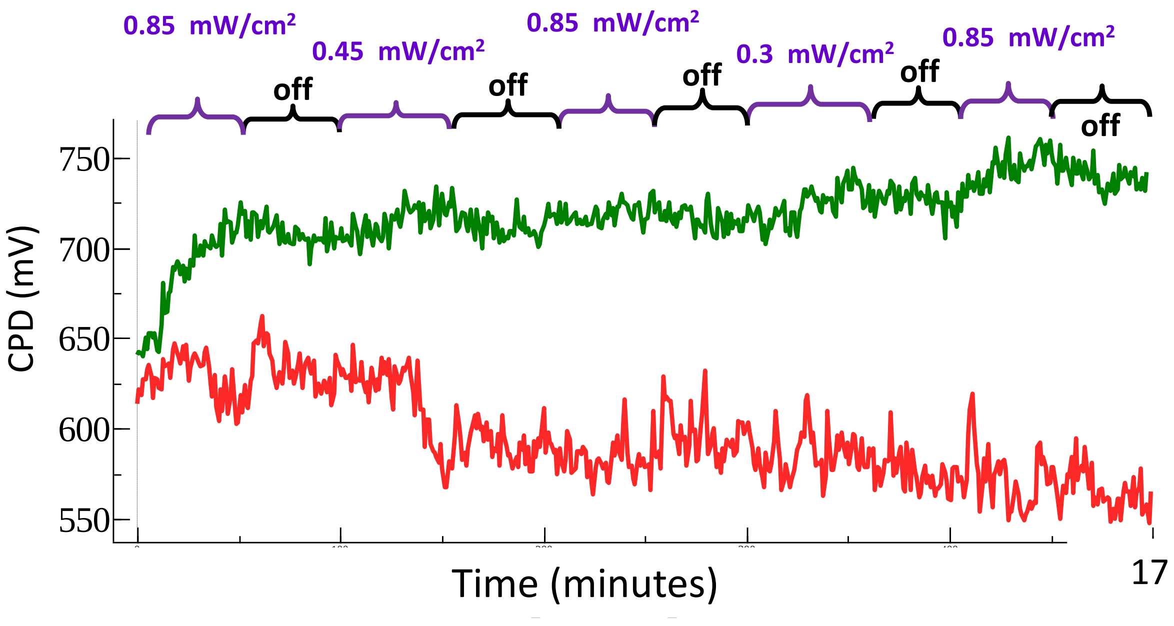

Figure S3. The charged trapped by the TiO_2 sample remains at the surface even when the light is off under an atmosphere of N_2 (green curve). When methanol is introduced with the N_2 (red curve) the V_{CDP} finally decreases, because methanol is a very effective hole scavenger.

We bubbled dry nitrogen through a sparger in a bottle containing methanol to saturate the nitrogen with methanol. The nitrogen flow rate was approximately 1 SCCM giving a flow rate of ~ 0.15 SCCM methanol at 20 °C. After 17 minutes a total of 0.1 mmol of Methanol would be delivered to the AFM. However, only a fraction of this methanol will adsorb and react at the surface. The vapor pressure of the methanol above the surface is expected to be 13 kPa.

4. Au NPs fabrication and deposition on the $TiO_2(110)$ surface. Au NP size analysis.

Pure gold nanoparticles (NPs) have been generated using the gas-phase approach[3]. In this work a so-called Multiple Ion Cluster Source (MICS) has been used. This method allows the fabrication of ligand-free NPs that can be soft-landed on surface in well-controlled atmospheres. The set-up has three independent magnetrons (one-inch diameter each) placed in an aggregation zone.[4-6] The pure gold target (99.95%) is magnetron sputtered in a well-controlled atmosphere (Ultra High Vacuum, UHV, base pressure in the low 10^{-9} mbar range) guarantying the purity of the extracted atoms and ions. Subsequently the atoms recombine producing the NPs that are extracted from the aggregation zone in the form of a beam thanks to differential pumping.[7] The NPs beam passes through an exit slit located at the end of the aggregation zone and is projected into a second UHV deposition chamber where the substrates are placed. The NPs soft-land on the substrates surfaces that are placed at normal incidence to the beam without deformation (i.e.: height equal to diameter).[8] The main advantages of the MICS are the production of ligand-free nanoparticles that can be deposited on any surface provided that it is vacuum compatible together with a narrow size distribution. Both the fabrication rate and size of the nanoparticles depend on the fabrication parameters; in our case we have used the following parameters: argon flux at gold magnetron = 30 sccm (standard cubic centimeters per minute), total argon flux = 100 sccm,[9] applied power = 8 W (U = 257 V, I = 30 mA) and distance between magnetron head and exit slit of aggregation zone = 70 mm. Prior to their deposition on $TiO_2(110)$ surfaces, the NPs have been deposited on silicon wafer substrates and characterized by Atomic Force Microscopy to adjust the desired NP size. The deposition times have been adjusted in order to produce deposits where the NPs are sufficiently isolated. NPs can be individually imaged by AFM as long as they are more than 40 nm apart, since 40 nm is the metallic tip average diameter (Figure S5).

Gas-phase synthesis of the NPs offers the possibility of a precise control of the fabrication conditions, generating NPs in a clean environment (as it works in ultra-high vacuum) resulting in ligand-free ultra-pure NPs. Thanks to these capabilities, gas aggregation sources are emerging as an alternative route for NPs fabrication of interest in catalysis. [10-12]

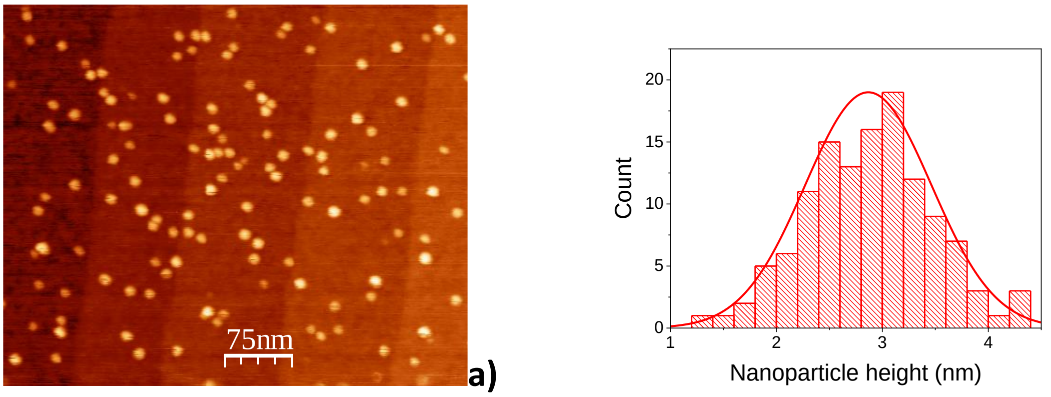

Figure S4. a) AFM image of Au NPs on TiO_2 and b) its size distribution. The particle height data were fit to a log-normal distribution resulting in an average nanoparticle diameter of 2.9 and a standard deviation of 0.5 nm (analysis of 115 nanoparticles).

5. TEM image of conductive tip.

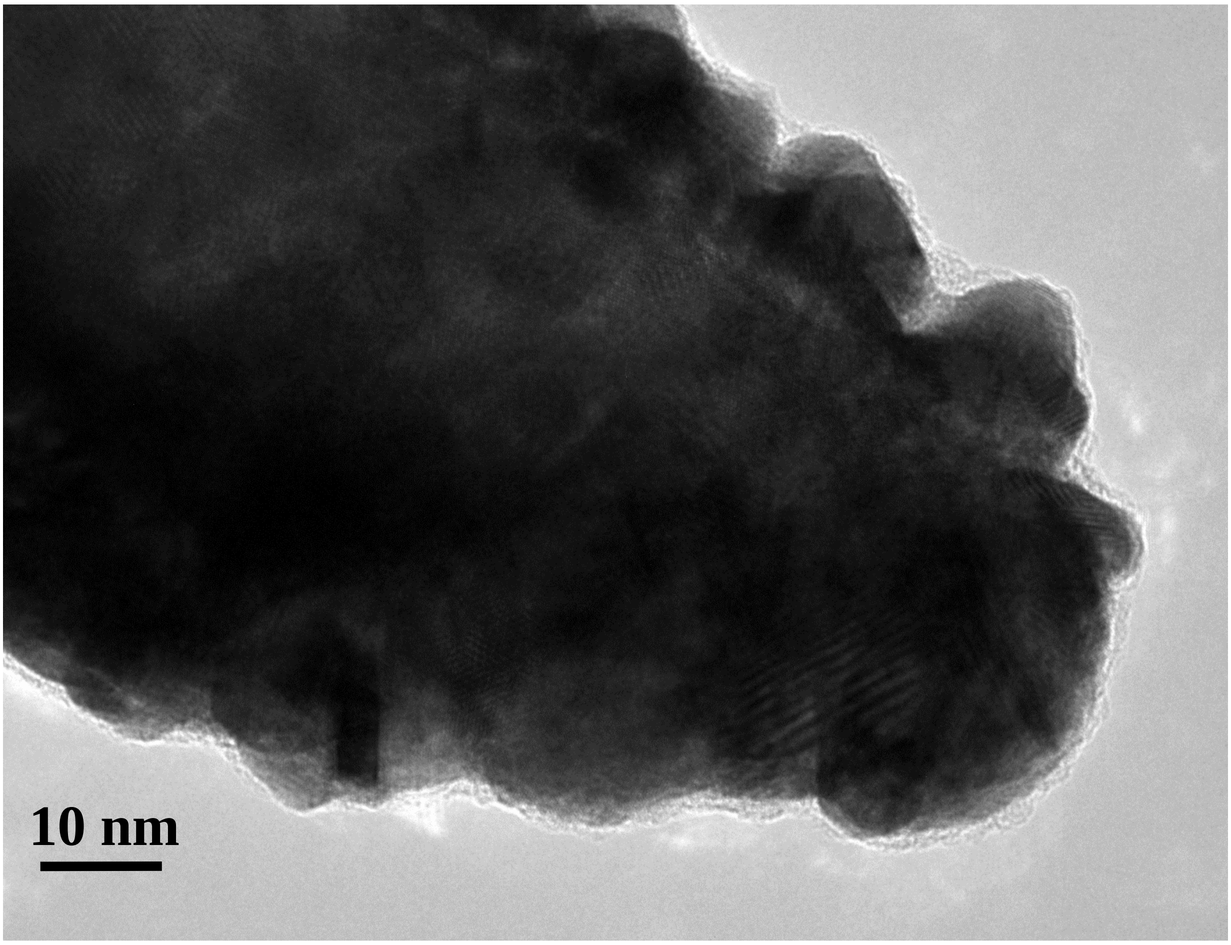

Figure S5. TEM image of a $PtIr_5$ coated Silicon Tip (ATEC-EFM-Nanosensors). The image was acquired in a JEOL 2100-F FETEM at 200 kV.

It should be noted that the spatial resolution of the V_{CPD} images in the text is roughly equivalent to the tip diameter, typically around 40 nm in our experiments.

6. Length of the Schottky junction depleted region for finite and small Au nanoparticle

A theoretical analysis of charge transfer in metal catalysts supported on a doped TiO_2 carrier has been performed by Ioannides et al.[13] The development of the theoretical model is based on the metal–semiconductor contact theory and was used to calculate the amount of charge transferred to supported metal crystallites, as a function of the electronic structure of the semiconducting support and the metal crystallite size

For a model with a finite interface, where a spherical metal particle of radius, r_M, is embedded in the semiconductor bulk (Figure S6), the length (D) of the depletion region can be determined as a function of the electrostatic potential barrier (V_{BB}), the donor concentration (N_d), the metal particle radius (r_M), the relative dielectric constant of the semiconductor (ε_r), the vacuum permittivity (ε_0), and the charge of the electron (e):

$$V_{BB} = \frac{eN_d}{\varepsilon_r \varepsilon_0}\left[\frac{(D + r_M)^2}{2} - \frac{{r_M}^2}{6} - \frac{(D + r_M)^3}{3r_M}\right]$$

For the materials used in the experiment of Figure 4 in the article: Work function of Au (W_{Au}) = 5.15 eV, W_{TiO2} = 4.2 eV, N_d = 1.39 10^{25} m^{-3}, ε_r = 120 y r_M = 1.6 nm, the length of the depletion region is 12.1 nm.

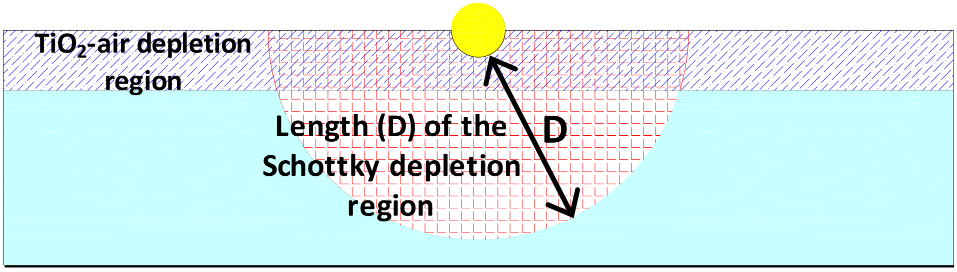

a)

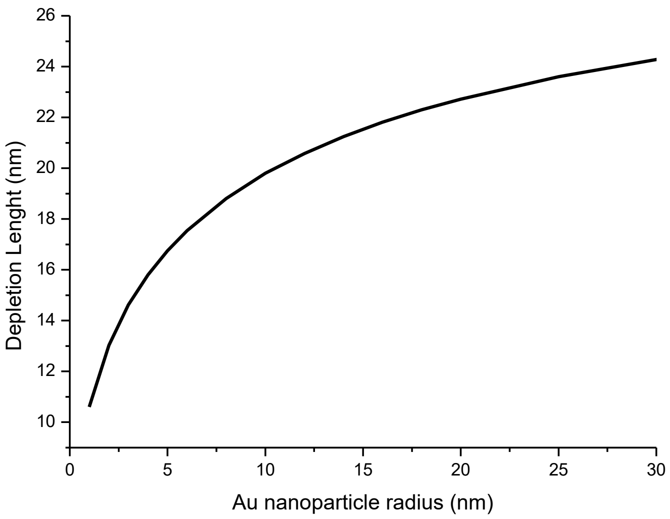

b)

Figure S6. (a) Scheme where the semiconductor-air depletion region is represented in dark blue and the semiconductor-metal NP depletion region is drawn in red squares. (b) Calculated depletion region length (D) vs. Au nanoparticle size for the materials and conditions of the experiment of Figure 4.

It is easy to calculate the N_b concentration (N_d) in $TiO_2(110)$ doped by 0.05% of the total weight of the crystal. It results in a concentration of Nb atoms of $1.39.10^{25}$ m^{-3}**.**

7. Theoretical calculation of the electrostatic interaction forces between tip and surface

The experiments discussed in the present work have been performed using AFM imaging in non-contact dynamic force microscopy, and using the gradient/frequency shift of electrostatic interaction to implement Kelvin Force Microscopy. As discussed previously[14], for these experimental conditions a realistic model of the tip can be described by two parameters, the radius R of the tip apex, simulated as a paraboloid, and an opening angle ϑ of cone representing the tip shaft, as shown in the inset of figure S7. Assuming a smooth transition (first derivative is continuous) at the point where the tip shape changes from parabolic to conical, this uniquely defines the overall tip shape.

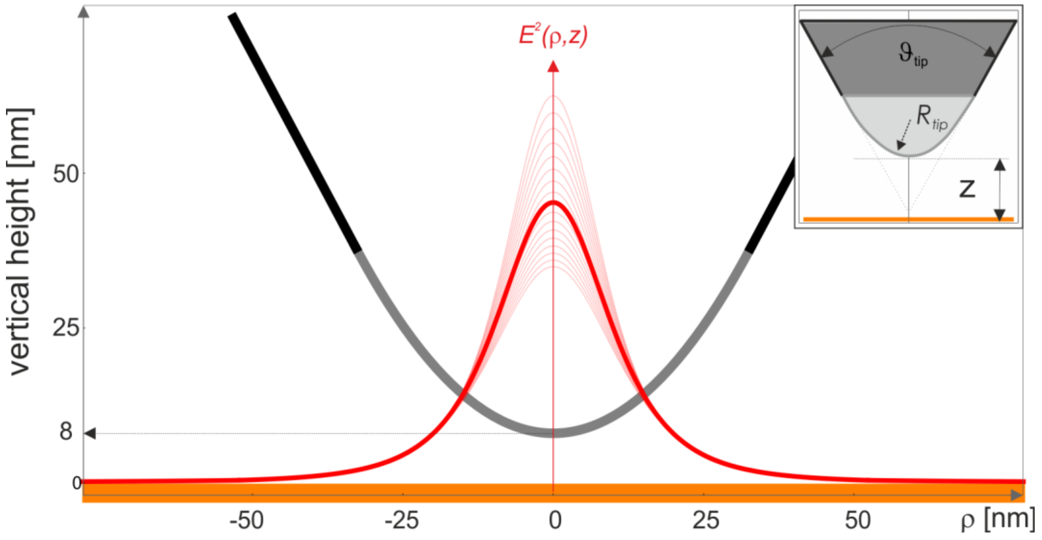

Figure S7. Tip profile (gray) and electric field distribution $E^2(\rho, z)$ using the experimental parameters obtained from the TEM image of figure S5: R_{tip} = 17.5 nm, tip cone angel ϑ_{tip} = 35°. The field distribution curves (red lines) were calculated for a set of tip-sample distances d from 6 to 10 nm. The thick red line corresponds to the field $E^2(\rho, z)$ for d = 8 nm, using arbitrary units in the ordinate. The abscise ρ is the distance of the tip apex from the origin in the polar coordinate system. Inset: tip geometry used for modelling the electric field on the surface assuming a parabolic shape for the apex transitioning to a mesoscopic cone shaft.

With the model for electrostatic forces described by Colchero et al.[14], the electrostatic field on a metallic surface is calculated for this tip-sample system and shown in figure S7 using the

parameters obtained from Figure S5: R_{tip} = 17.5 nm, ϑ_{tip} = 35° and a tip-sample distance of about 8 nm. The thick red line corresponds to d=8 nm, and the thinner lines correspond to field distributions for d ranging between 6-10 nm, in steps of 0.5. The electrostatic field intensity can be interpreted as the tension (units of pressure, N/m^2) generated by the field lines connecting (opposite) charges on tip and sample.

5.1. Electrostatic lateral resolution

As discussed in more detail elsewhere,[14] the (total) surface integral over this field intensity on the sample surface gives the total electrostatic force gradient:

$$F'(z) = \frac{\varepsilon_0}{2} \int dS \frac{d}{dz} E^2(x, y; z) \qquad \text{(S1)}$$

Therefore, the electrostatic field intensity can be interpreted as the aperture function for electrostatic measurements, which essentially defines resolution in KPFM mode, in good agreement with our experiments.

As can be observed from the Figure S7, at a distance of 10 nm from the symmetry axis (X), the field intensity $E^2(\rho, z)$, decays to about 50% of its maximum. We can also observe that although the maximum of the field intensity varies significantly with distance (as does the total electrostatic force), the width of these curves –of the order of the tip radius- stays essentially the same, implying that the lateral resolution is similar for the range of tip-sample distances (z) shown. We attribute this to the fact that for all the field distributions shown in Figure S5 tip-sample distance is (significantly) smaller than the tip radius. For z > R however, the field distributions become wider, leading to a reduced electrostatic resolution, as expected and as discussed previously[14].

5.2 Surface potential difference between two conductors with different size: bulk and Nano-size.

The interaction force gradient induced by the lever and the cone´s tip is strongly reduced in the range of distances relevant in the AFM experiments. Only the apex of the tip contributes to the total force.[14] For distances smaller than the radius of the tip ($z < R_{tip}$):

$$F'(z) \cong F'^{apex}(z) \cong -\pi\varepsilon_0 V^2 \frac{R_{tip}}{z^2} \qquad \text{(S2)}$$

By using the force gradient as signal source for the interaction in KPFM, local contact potential differences can be obtained.[14]

Since

$$F'(z) = \frac{\pi\epsilon_0}{4} C''(z) V^2 \qquad \text{(S3)}$$

the primes indicating derivatives, we obtain for the second derivative of the capacitance:

$$C''(z) \propto \frac{R_{tip}}{z^2} \qquad \text{(S4)}$$

The total potential V between tip and sample includes the contact potential difference between tip and sample (V_{CPD}) and the external potential applied ($V_{bias}(x,y)+V_{ac}\sin\omega_e t$); the force gradient signal that is used for the KPFM detection ($1\omega_e$ component of the electrostatically induced tip-sample interaction) is then:

$$F'(z) \cong C''_{apex}(z)[V_{CPD} - V_{bias}(x,y)]V_{ac}\sin\omega_e t \qquad \text{(S5)}$$

Where $V_{ac}\sin\omega_e t$ is the ac component of the bias potential externally applied to the tip. In KPFM the V_{CPD} is obtained by finding the value of V_{bias} for which the force gradient vanishes.

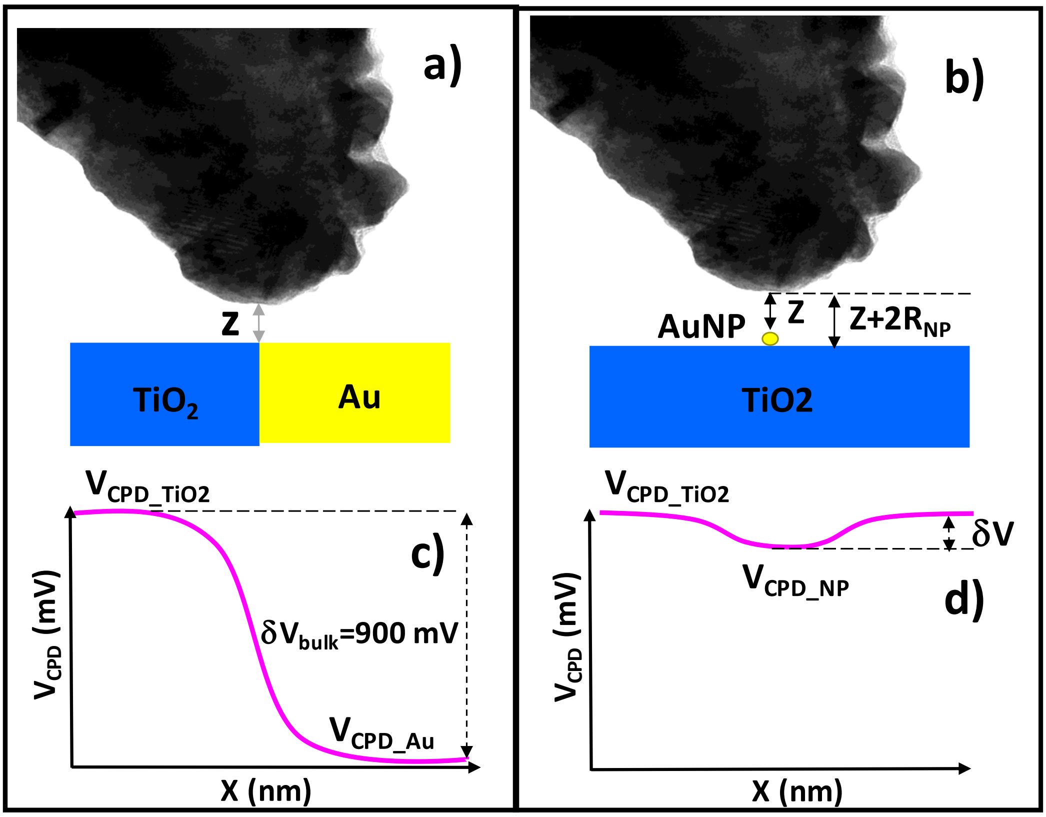

Figure S8. Two distinct schemes in which KPFM is performed on different samples consisting of bulk TiO_2 in contact with bulk Au (a), and of a Au NP on a TiO_2 surface (b). The V_{CPD} profiles c) and d) correspond to the a) and b) layouts respectively.

Figure S8a schematically illustrates the V_{CPD} profile obtained by KPFM for the case of a sample consisting of bulk highly doped TiO_2 and bulk gold. The contact potential difference (V_{CPD}) far from the junction would show a CPD between both materials of about 900 mV, equal to the difference in their work function values (4.2 eV for Nb doped TiO_2 (110) and 5.1 eV for gold). Figure S8b corresponds to the geometry of the Au NP on the TiO_2 sample. The difference in V_{CPD} between the TiO_2 surface and the area above the Au NP (δV) will be smaller than in the previous geometry since the tip feels the effect of the small Au NP and also the larger TiO_2 surface area around the Au NP. Note that the force (and force gradient) induced by electrostatic interaction is due to the field between tip and sample given by $E^2(\rho,z)$, as shown in Figure S7. To calculate the

experimental surface potential difference δV expected for the case of the small Au NP, the two contributions: tip-Au NP (at a distance of Z), and tip-TiO_2 surface below the Au NP (at a distance of of Z+2R_{NP}), should be included. This can be interpreted as two capacitances in parallel. The AFM tip then "feels" a weighted effective potential difference V_{CPD_NP}, where these two contributions add to V_{CPD_NP}, weighted by the corresponding capacitance second derivative:

$$V_{CPD\_NP} = \frac{C''_{tip\_TiO2} V_{CPD\_TiO2} + C''_{tip\_AuNP} V_{CPD\_Au}}{C''_{tip\_TiO2} + C''_{tip\_AuNP}} \quad \text{(S6)}$$

By applying equation S4:

$$C''_{tip\_TiO2} = \frac{R_{tip}}{(z+2R_{NP})^2} \quad \text{(S7)} \quad \text{and} \quad C''_{tip\_AuNP} = \frac{R_{NP}}{(z)^2} \quad \text{(S8)}$$

and substituting S7 and S8 in S6, with the experimental values: Z = 10 nm, R_{tip} = 18.5 nm and R_{NP} = 1.5 nm, δV =V_{CPD_TiO2} - V_{CPD_NP} equals 120 mV. Therefore, a significant reduction in surface potential difference between TiO_2 and Au (Figure S8b) of 87% is expected with respect to the difference in the bulk case (Figure S8a). However, the experiment shows an even lower value for this difference (12 mV, Figure 4a). The depletion region around the Au NP could be playing a role in further decreasing the difference. In addition, the presence of image charges also reduces the electrostatic interaction force, as explained in the next section.

8. Theoretical model for calculating electric charge inside the nanoparticle

By simplifying the modeling of the elements that play a role in the electrostatic interaction between tip and surface, an analytic expression for the electrostatic forces and charges can be obtained. The following approximations have been implemented (Figure S9a):

1. A spherical conductive tip with a radius R_{tip} and a charge Q.

2. A semi-infinite grounded conducting sample.

3. An infinite plane with a charge density σ added on the sample surface in order to simulate the surface contact potential.

D is the tip-sample scanning distance. A DC as well as an AC voltage are externally applied to the tip during KPFM operation:

$$V_{tip} = V_{dc} + V_{ac}\sin(\omega t) \qquad \text{(S9)}$$

The potential applied to the tip in addition to the contact electric field generated by the surface charge density σ, induce an electrostatic force between tip and sample. The force has been simulated with the Generalized Image Charge Method (GICM),[15] which uses image charges to model the charge that appears in all surfaces and cancels the electric field inside the metallic elements.

When the tip scans over the bare sample (with no NP on it, Figure S9a), the tip initially is electrostatically equivalent to that of a charge Q inside which is related to the electrostatic potential applied to it. By assuming a sphere as the shape of the tip, the value of Q can be calculated:

$$Q = 4\pi\varepsilon_0 R_{tip} (V_{dc}+V_{ac}\sin(\omega t)) \qquad \text{(S10)}$$

A second contribution to the tip charge is the image charge $-q_\sigma$ which originates from the constant electric field generated by the surface charge density σ. Both tip charges (Q and $-q_\sigma$) induce image charges inside the conducting sample (q_σ and -Q).

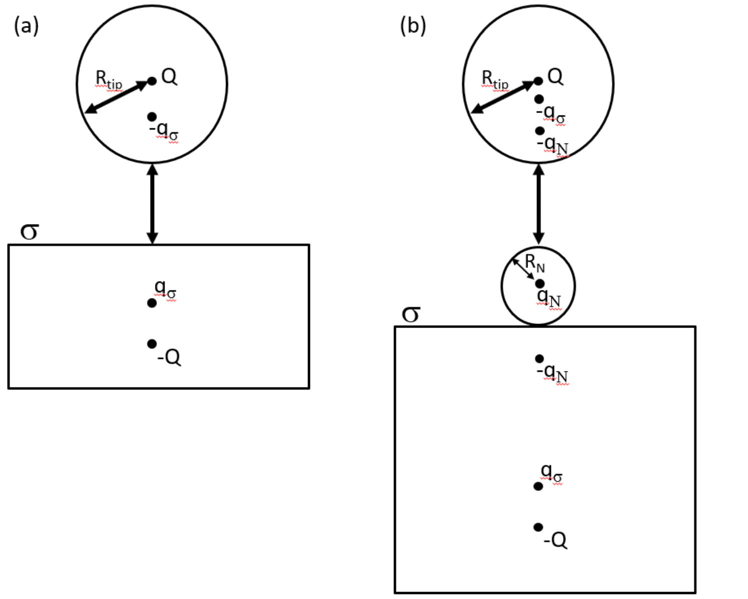

Figure S9. For the electrostatic modelling we assume a spherical conducting tip scanning over a semi-infinite grounded conducting bare sample (a) and over a metal nanoparticle placed on the sample (b). Q: charges generated by the external voltage applied in KPFM; $-q_{\sigma}$: charge on the tip originated by the interaction with the surface charge density σ

The electrostatic interaction force on the tip (F_{tip}) can be calculated by including all the interactions between the charges inside the tip and all the charged elements on the sample. F_{tip} presents only a vertical component:

$$F_{tip} = \sum_{i=1}^{Ne} F(Q_i) + \sum_{i=1}^{Ne} F(-q_{\sigma i}) \qquad \text{(S11)}$$

Where Ne is the number of charge elements on the conducting sample. If only the first image charge obtained from the GICM is taken into account, then N = 3 (corresponding to σ, -Q and q_σ inside the sample). Combining equations 2 and 3, we obtain three contributions to the electrostatic force: one that is independent of ω, a second one that is proportional to sin(ωt) and a third one

proportional to sin(2ωt). In KPFM, we focus on the signal proportional to sin(ωt) since the bias voltage applied to the tip (Vdc) is the value that cancels this force contribution.

To model the electrostatic force on the tip when the tip scans above a metal nanoparticle placed on the sample surface (Figure S9b), an additional term must be included. The extra contribution originates from the charge trapped in the nanoparticle (q_N), as shown in Figure S9b. In the simplest scenario there will be 3 charged contributions inside the tip and 5 charged elements on the surface. The interaction force, in this case, will be:

$$F_{tip} = \sum_{i=1}^{Ne} F(Q_i) + \sum_{i=1}^{Ne} F(-q_{\sigma i}) + \sum_{i=1}^{Ne} F(-q_{Ni}) \qquad \text{(S12)}$$

It is worth noting that in order to nullify this force component, the voltage $V_{dc'}$ applied to the tip must be different from the previous case (V_{dc}), since now more contributions to the force are present. As can be observed in Figure S9b, there are two contributions to the force: q_N (the charge on the nanoparticle) and $-q_N$ (its image charge inside the conducting sample) that are very close to each other because the nanoparticle radius (R_N) is very small compared to R_{tip}. Since these two contributions are proportional to q_N with opposite sign and very close to each other, in a standard KPFM setup, the electrostatic interaction force that these two charges will exert on the tip will be up to 5 times smaller than the force theoretically felt by the tip when assuming only the original q_N.

Assuming an additional simplification to the model, where we consider $R_{tip} >> R_N$ and $R_{tip} >> D$, we can obtain q_N from the following expression:

$$q_N = \frac{2\pi\varepsilon_0 R_{tip}}{(D+R_{tip})^2}(V'_{dc} - V_{dc})\left(\frac{1}{(D+R_{tip}+R_N)^2} - \frac{1}{(D+R_{tip}+3R_N)^2}\right)^{-1} \qquad \text{(S13)}$$

For the values of the experiment (R_{tip}=18.5 nm, R_N= 1.5 nm, D= 10 nm), the charge inside the nanoparticle obtained with our model is 5.25 10^{-20} C when we do not apply any additional

illumination (V'dc-Vdc=8mV) and 1.64. 10^{-19} C when the sample is illuminated (V'dc-Vdc=25mV).

9. Au NP on TiO_2 CPD image of large areas.

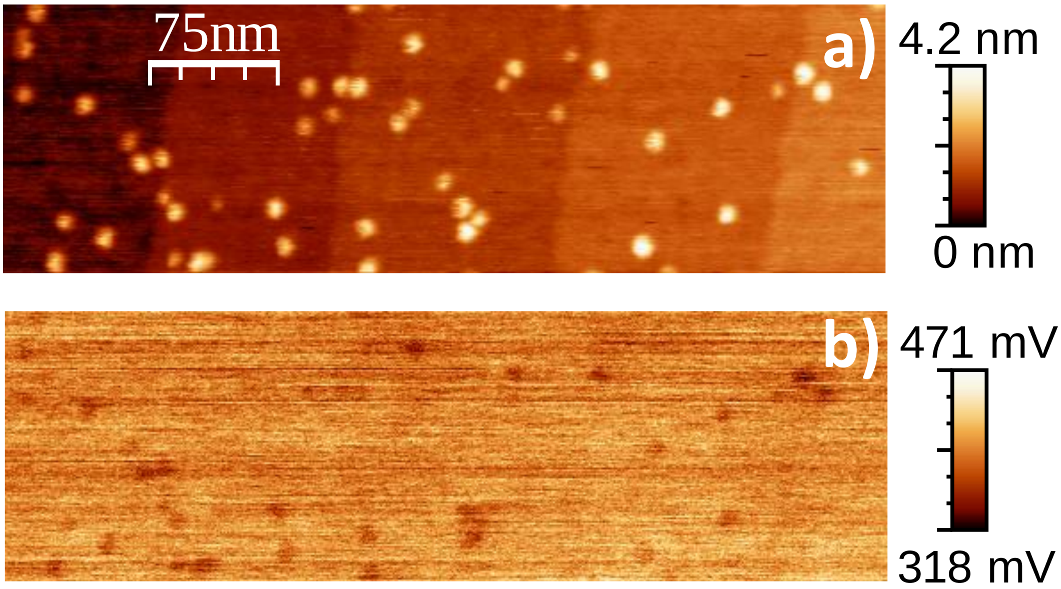

Figure S10. Topographic (a) and CPD image(b) of Au NPs on TiO_2. The CPD is lower on the Au NP, as expected when a large work function material, such as Au, is in contact with a lower work function one (TiO_2): electrons transfer from the low work function material (TiO_2) to the high work function material (Au). Au NP that are smaller than 3 nm in diameter generate weak contrast that may not be observable. This is due to the comparably large size of the tip relative to the size of the NP.

10. Topography, CPD and Resonance Frequency images of an Au NP on $TiO_2(110)$ by means of PA-KPFM.

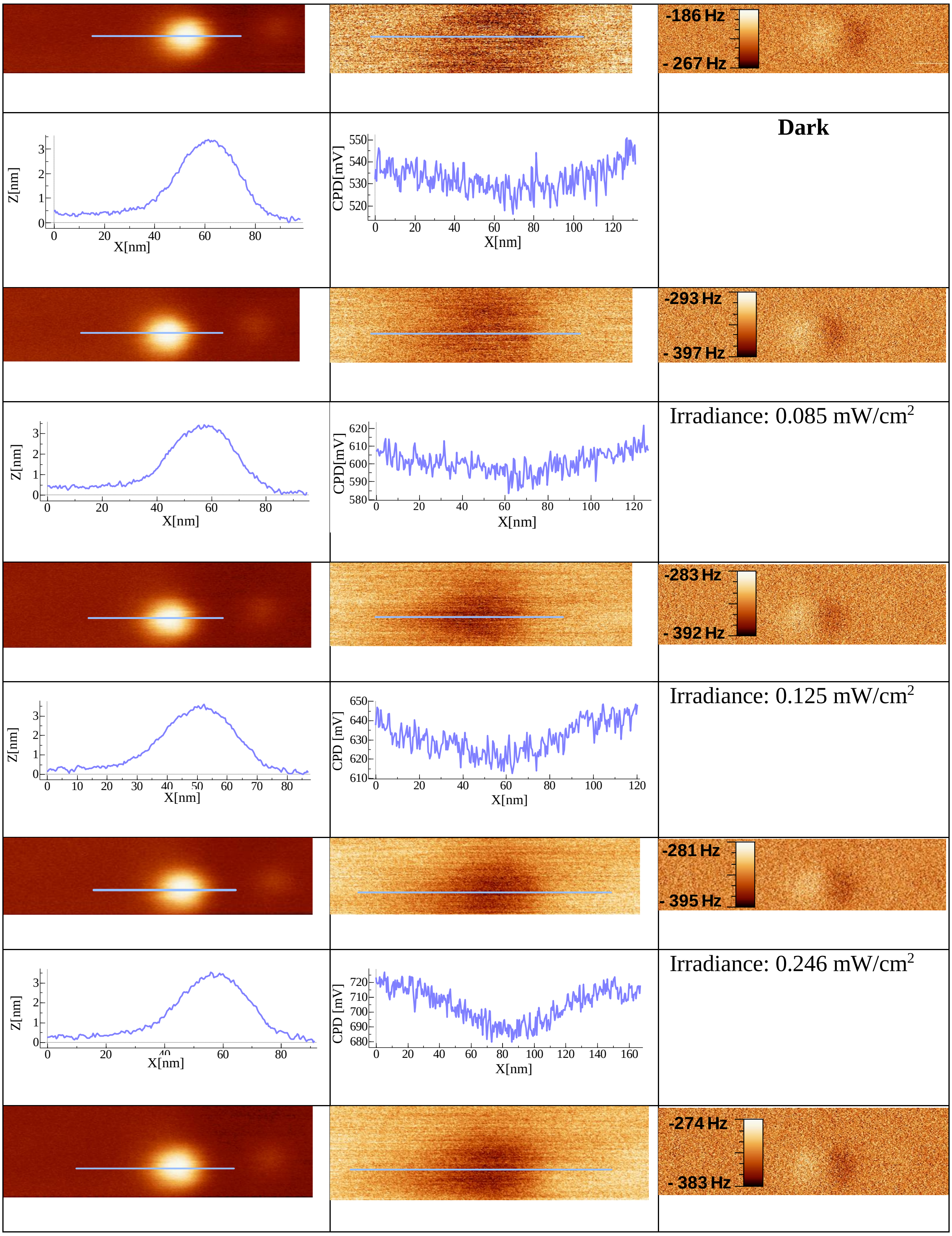

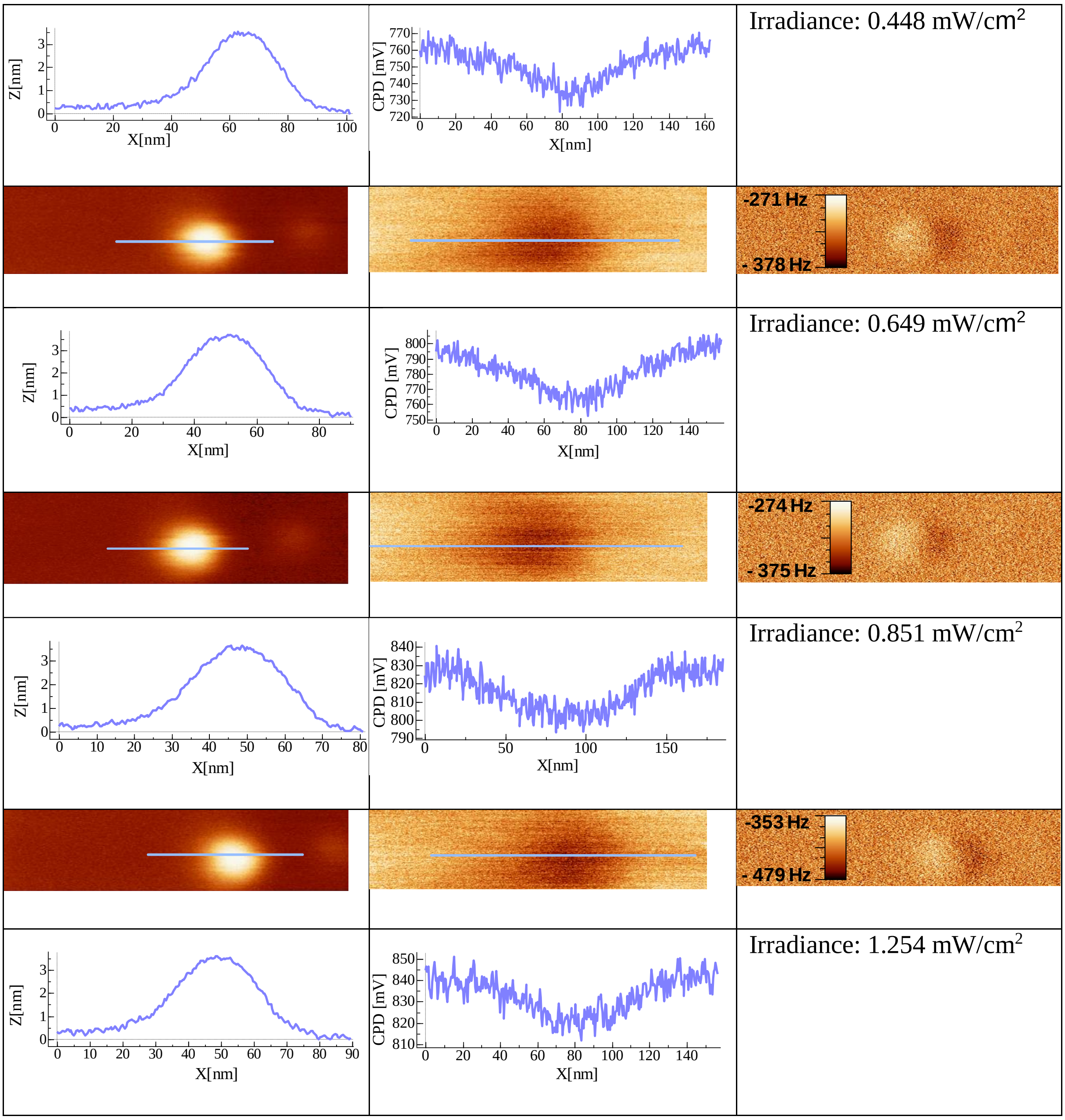

Figure S11. Ligand free Au NP on Nb doped TiO_2(110) monocrystal. Left column: Topography and marked profile. Middle column: CPD and marked profile. Right column: Resonance Frequency. The value of the resonance frequency in the images is close to the value of the free cantilever resonance frequency and thus the contrast in the images is weak. Thus it can be concluded that the scanning of the images was performed out of contact. If tip-sample contacts happen during the imaging process, when operating the AFM in air, liquid necks could form, even at low relative humidity values. The formation and rupture of liquid necks induces a delay in the oscillation frequency of the cantilever which is reflected in the resonance frequency image.

11. Evaluation of carbonaceous molecular species on TiO_2 (110) by means of sum frequency generation spectroscopy.

In order to evaluate the self-cleaning efficiency of 365 nm wavelength irradiation on TiO_2 (110) we have performed sum frequency generation (SFG) spectroscopy experiments. SFG is a powerful method of studying molecules at surfaces. It is a surface-specific technique which intensity is enhanced when the frequency of the infrared beam is in resonance with an SF-active molecular vibrational mode.

We used a picosecond laser system to generate a 1064 nm near-infrared light with repetition rate of 20 Hz. A Laser Vision optical parametric generator and amplifier system converts the 1064 nm to a visible 532 nm beam and a mid-infrared beam ranging between 2200 cm^{-1} to 4000 cm^{-1}. Sum frequency generation is achieved when the visible and infrared beams overlap spatially and temporally on the sample. We collected the sum frequency signal reflected from the sample's surface (reflectance mode). The beam orientations in all the SFVS experiments were: 45° for the 532 nm beam, and 56° for the mid-infrared beam, with respect to the perpendicular plane (reference plane). The polarization combination used in this work was SSP (S-polarized light for the SF output and for the input visible beam, and P-polarized light for the input infrared beam).) The measurements were performed in air. The temperature was 296 K and the relative humidity 40%.

Fig. S12a) shows a spectrum of the TiO_2 (110) sample before UV irradiation. The presence of CH_2 and CH_3 can be observed in the regions of 2850, 2930 and 2940 cm^{-1}. Fig. S12b) combines the spectra shown in a) with the one obtained for1 ML of OTS. From the comparison it can be inferred that the contamination density on TiO_2 is very low.

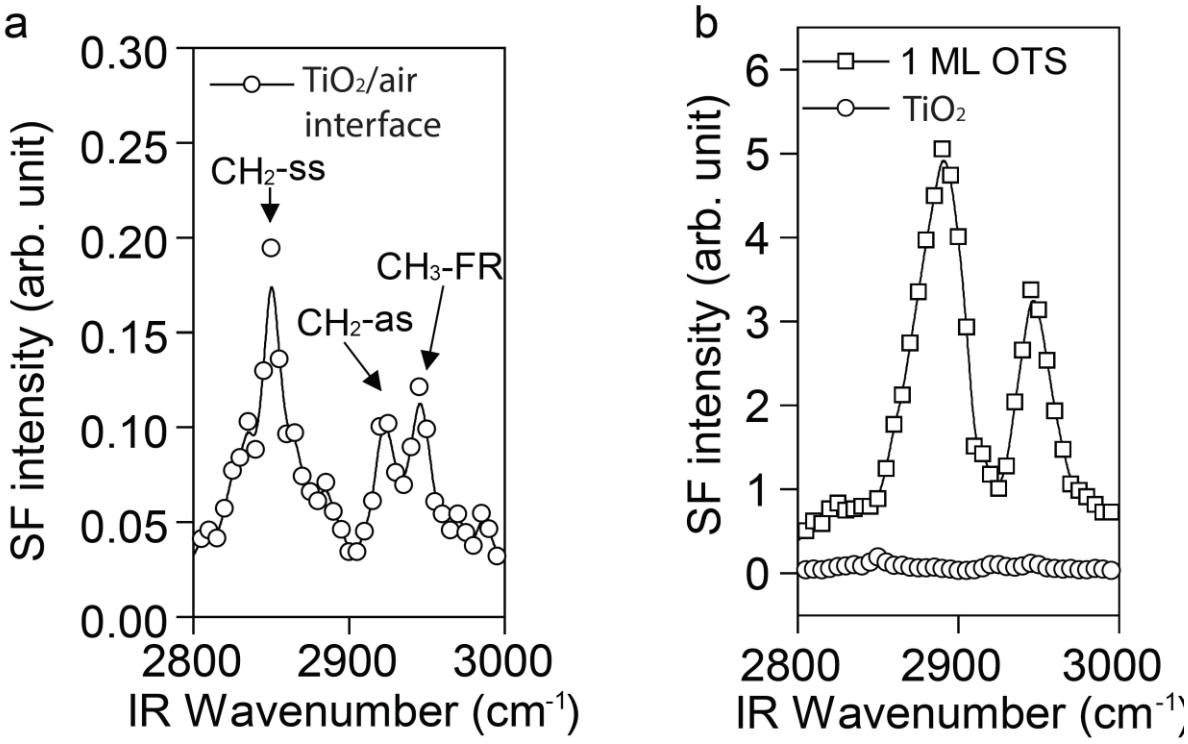

Figure S12 a) SFG spectrum of non-irradiated TiO_2 (110). The three observed peaks correspond to the methylene stretch mode (2850 cm^{-1}), the methylene antisymmetric stretch mode (2930 cm^{-1}) and the methyl group (Fermi Resonance, 2940 cm^{-1}). **b)** Comparison of the SF intensity between the carbonaceous surface peaks from TiO_2 and 1 monolayer of OTS.

Figure S13a) shows 6 SF spectra taken before irradiation (t= 0) and during irradiation with 365 nm wavelength light, at increasing exposure times. After 1 hour of UV illumination, the carbonaceous species are removed from the surface. After keeping the sample 1-2 hours in the dark, the presence of carbonaceous components can be observed again (Fig. S13b).

Since the sample was exposed to 365 nm wavelength light irradiation before the KPFM experiments and the whole experiment did not last for more than 45 minutes, we do not expect carbonaceous species from ambient contamination to play an important role in the present work results.

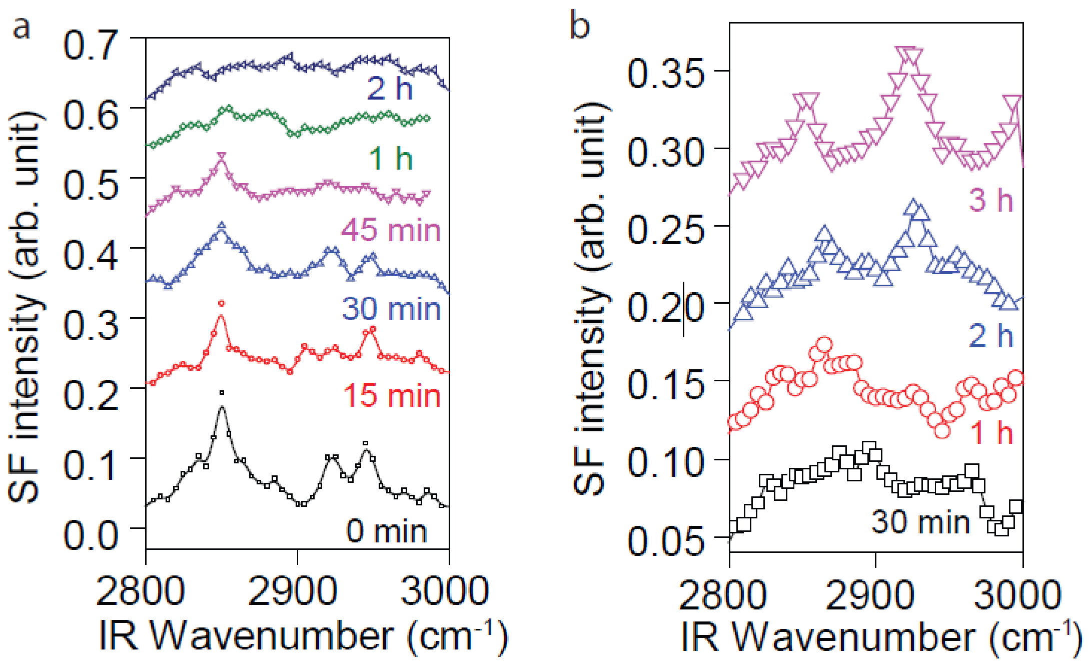

Figure S13 a) SFG spectra before illumination (0 min) and after 365 nm of wavelength light illumination (0.5 mW/cm^2) at increasing time exposure. Carbonaceous species are removed from the surface after 1 hour of illumination. **b)** Spectra taken in darkness after the experiment shown in a). After 1-2 hours with the light off, peaks corresponding to CH species start being observed again.

SFG spectra were also performed in order to probe the presence of a water layer on the sample surface. Figure S14 shows a SFG spectrum of TiO_2 in the region where the OH vibrational modes of water are expected to be found: a well-defined, narrow peak at 3700 cm^{-1}, conventionally assigned to the free OH mode of the adsorbed water molecule, and two broad peaks centered around 3150 and 3400 cm^{-1}, commonly assigned to hydrogen-bonded OH. It is well-known that there is adsorbed water on an oxide surface, but because there is no peak in the hydrogen-bonded OH stretching vibration region, it can be concluded that no liquid water layer but isolated water molecules exist on the surface in the present case. Thus, a low quantity of water molecules could be adsorbed on the TiO_2 surface, but without forming a liquid layer.

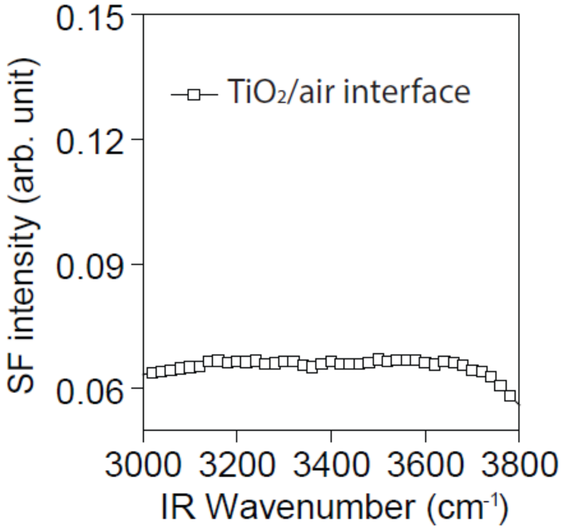

Figure S14. SFG spectra before irradiation in the region of the OH vibrational modes of water.

12. Photoelectrochemical characterization

For the H_2 evolution experiments, current density (at dark and under illumination) and voltage dependence were measured with a potentiostat-galvanostat PGSTAT204. A gas chromatography coupled to the photoelectrochemical cell was used to quantify the photogenerated hydrogen.

Photovoltage in electrochemical experiments also follows also a logarithmic behavior vs. irradiance (Figure S15).

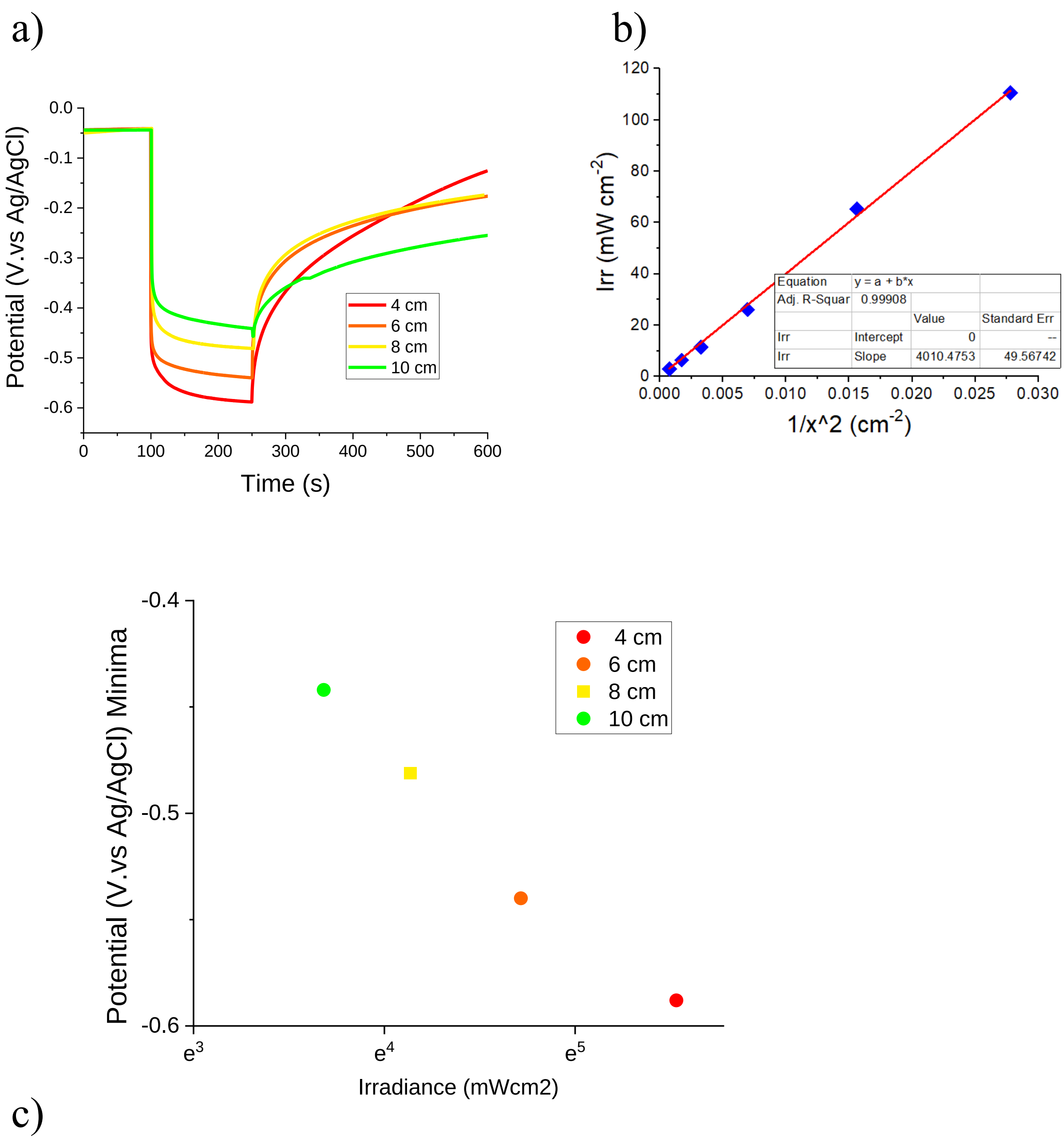

Figure S15 (a) Irradiance dependence Photovoltages vs. time in a photoelectrochemical cell. (b) Calibration of light irradiance with sample distance. The light irradiance was varied by changing the distance between the light source and the sample. (c) Photovoltages vs. Irradiance.

By electrochemical impedance spectroscopy (EIS) it is also possible to determine other parameters related with the double layer formed in the semiconductor-electrolyte interface. By making a fit of the different diagrams obtained with the different impedance values, it is possible build an equivalent electrical circuit and obtained resistance and capacity values related with the charge transfer across this double layer and depletion layer in the semiconductor- electrolyte interface. Figure S16 shows a Nyquist plot obtained at 0.4V vs Ag/AgCl under dark and illumination conditions. Charge transfer resistance decreases on both types of samples when exposed to illumination or when a potential is applied. The samples with Au NPs present lower resistance than those without NPs, both in the dark and under illumination. Therefore, the presence of Au NPs improves the conductivity of electrons through the TiO_2.

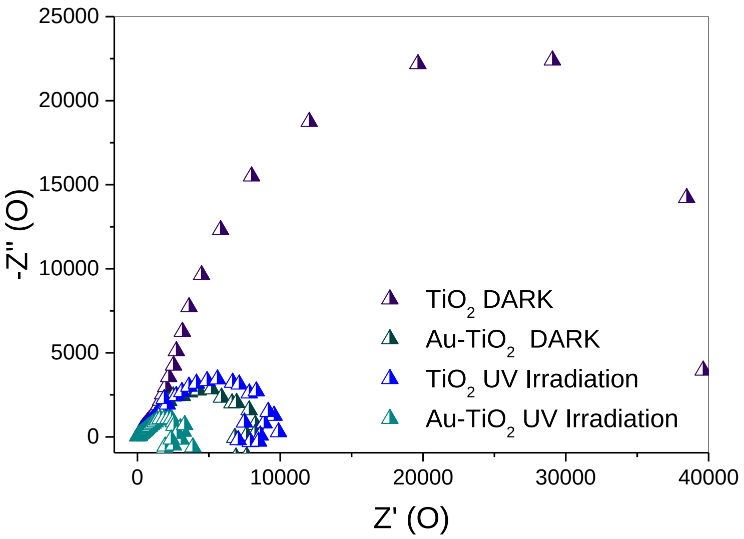

Figure S16. Nyquist plots obtained by Electrochemical Impedance Spectroscopy (EIS) of the doped TiO_2 crystals with and without Au NPs under 0.4V vs Ag/AgCl.

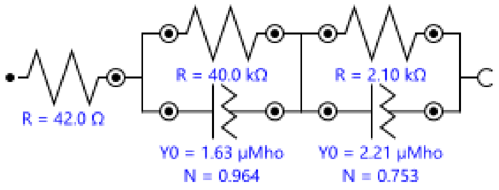

a)

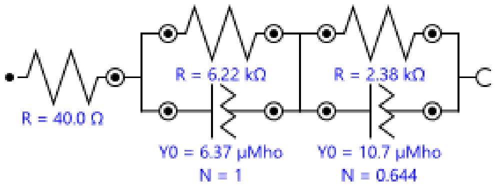

b)

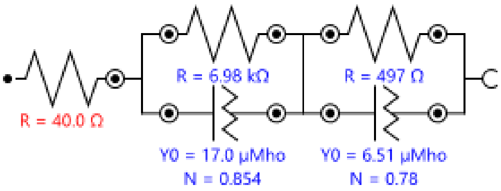

c)

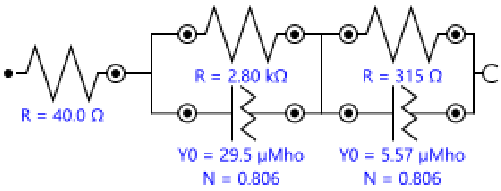

d)

Figure S17. Electrical equivalent circuits for TiO_2 crystals, with (c and d) and without (a and b) Au NPs, in dark (a and c) and under illumination (b and d).

Energy band edges of a semiconductor can be experimentally estimated from the determination of the flat band potential (VFB). This value can be determined by Electrochemical Impedance Spectroscopy (EIS) and using the Mott–Schottky equation.[16] The flat band potential serves to set the Fermi level of a semiconductor.[17]

In order to determine the space charge layer capacitance, AC modulated cyclic voltage scans from 0V to -1.2V (vs Ag/AgCl), at 400 kHz, were performed, at dark conditions. The capacitance of the space charge layer is calculated by assuming:

$$1/(wZ_{im}) = C_{SC}$$

where w is angular frequency and Zim is the imaginary part of complex impedance. The dependence of C_{SC} on bias potential is described by the Mott-Schottky equation:

$$\frac{1}{C_{SC}^2} = \left(\frac{2}{\varepsilon_{SC}\varepsilon_0 N_{D/A} e_0 A}\right)\left([V - V_{fb}] - \frac{k_B T}{e_0}\right)$$

where C_{SC} is the measured differential capacitance per area unit, e_0 is the elementary charge, ε_{SC} is the dielectric constant, ε_0 is the electrical permittivity of vacuum, $N_{D/A}$ is carrier density. Therefore, from the $1/C_{SC}^2$ vs. V plot, VFB can be easily obtained by the interception with the x-axis. The term k_BT/e_0 usually can be neglected because of its low value.

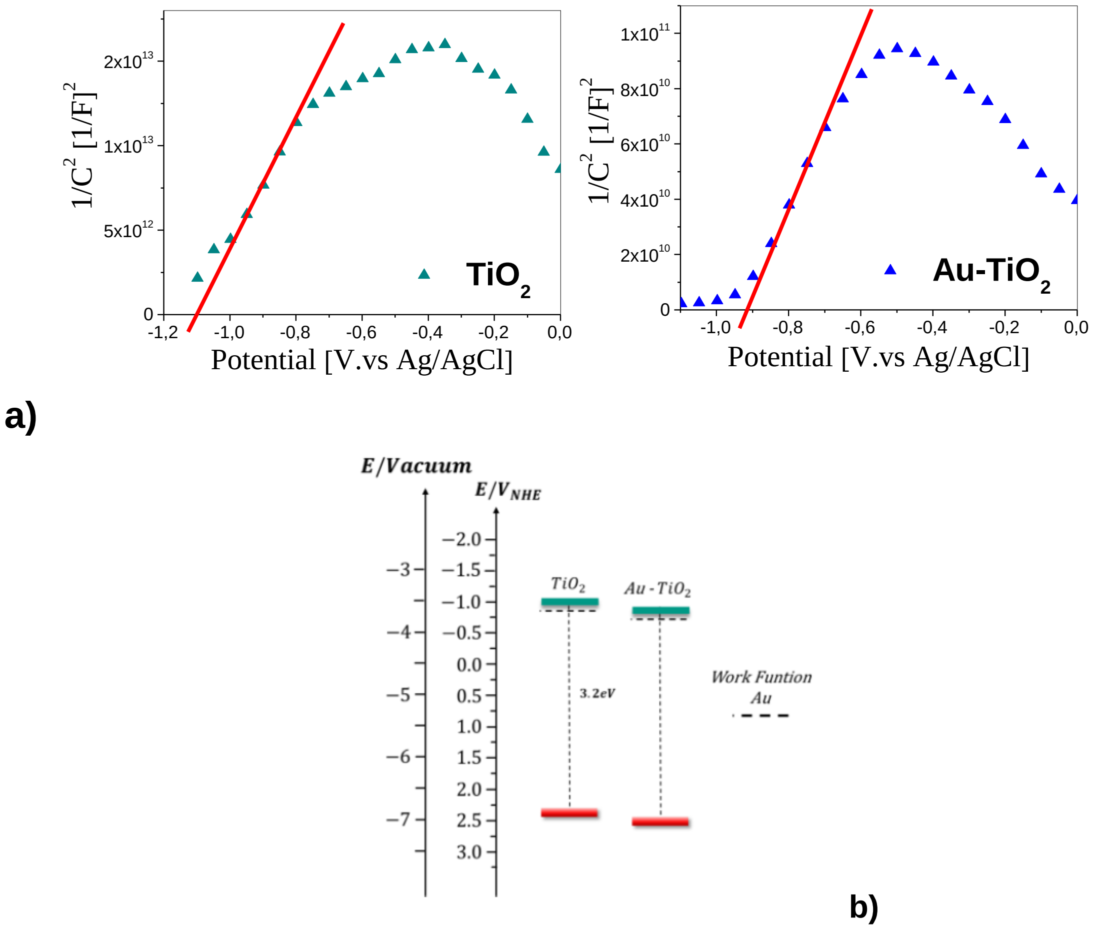

Figure S18. a) Mott-Schottky plots of TiO_2 monocrystal with and without Au NPs on the surface, at 400 Hz. The fits exclude the first low potential points since those are close to the aqueous electrolyte detection limit. b) Energy band diagram of monocrystal with and without Au NPs.

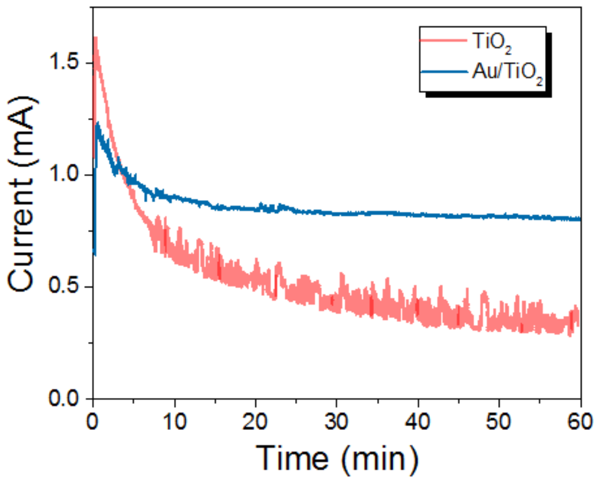

Figure S19. Photocurrent of TiO_2 and AuNPs on TiO_2 under UV irradiation at 0.7 V (vs. Ag/AgCl) during one hour.

13. AFM modification for light implementation and light calibration

A fiber-coupled LED (M365FP1, Thorlabs Inc.) was used for illuminating the sample (wave length 365 nm). Small modifications were needed in order to introduce the light beam in the vertical optical path of the Cypher ES (Asylum Research, Oxford Instruments) AFM head:

1. The view module has to be lifted 5 cm to allow for space underneath for a small positioning stage and the beamsplitter attached to it. The fiber is placed at the center of the stage. By means of the stage micrometer screws, the position of the spot on the sample can be relocated. The UV beam reaches the beamsplitter from the side and deflects it 90 degrees down towards the cantilever and sample.
2. After the 90 degrees beam splitter deflection, the beam goes through the AFM head dichroic hot mirror and from there to the objective lens. Since the objective was designed for fluorescence operation, a good optical performance is achieved at 365 nm of wavelength.
3. After the beam reaches the cantilever and sample, it gets reflected back up towards the view module. The camera at the view module is not sensitive to UV, therefore, in order to be able to inspect the placement of the UV spot on the surface and its diameter, a piece of the UV/VIS Detector Card VRC1 (Thorlabs) was placed instead of the sample. UV incident light generates visible light emission. Figure S20 shows an image of the beam spot at the surface of the detector card obtained with the View Module, when focusing on the sample (a) and when focusing on the cantilever (b).

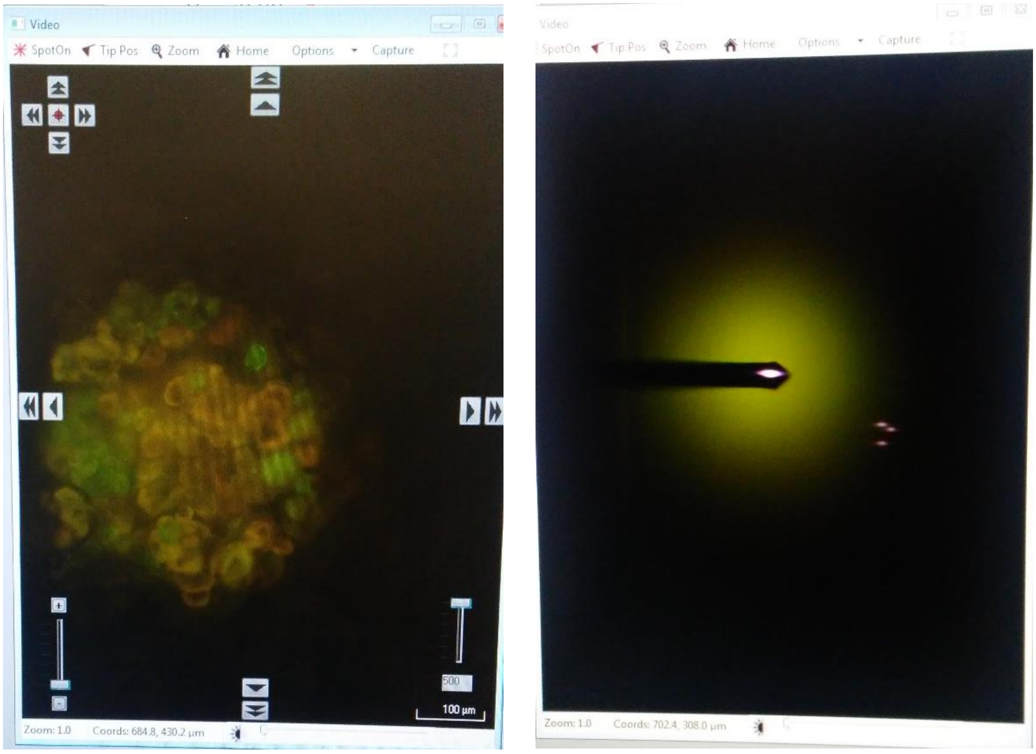

(a) (b)

Figure S20. Images from the View Module when the camera is focused on the sample surface: a piece of UV detector card (VRC1, UV/VIS Detector Card: 250 to 540 nm, Thorlabs) (a), and when focused on the cantilever. The tip of the cantilever protrudes at the end of the cantilever (ATEC-EFM, Nanosensors), so neither the cantilever, nor the tip shadows the surface from the UV light.

The size of the spot is around 300-500 microns in diameter. Since the tip of the cantilever extends out from the edge of the cantilever (ATEC-EFM cantilevers, Nanosensors), the surface underneth the tip is not shadowed from the UV light.

We have experimentally measured the SPV of the metal cantilever. We performed KPFM on samples which do not adsorb UV light (i.e., graphite), while turning on and off the UV light. Since the CPD measures the difference in electric contact potential between the tip and the sample, if the light had any effect on the tip surface, it would be noticed on the CPD image. Results show that the CPD value is the same in the dark and under light exposure in all the cantilevers used here, confirming that the metal coating of the tip is intact. TEM images of the metal tips also show an unbroken metal layer (Figure S5).

To calibrate the light intensity (Figure S21), we replaced the sample by the sensor S120VC, from Thorlabs. For each value of the UV LED current we recorded a light power measurement. The irradiance value was obtained by dividing the light power by the area of the UV spot on the sample.

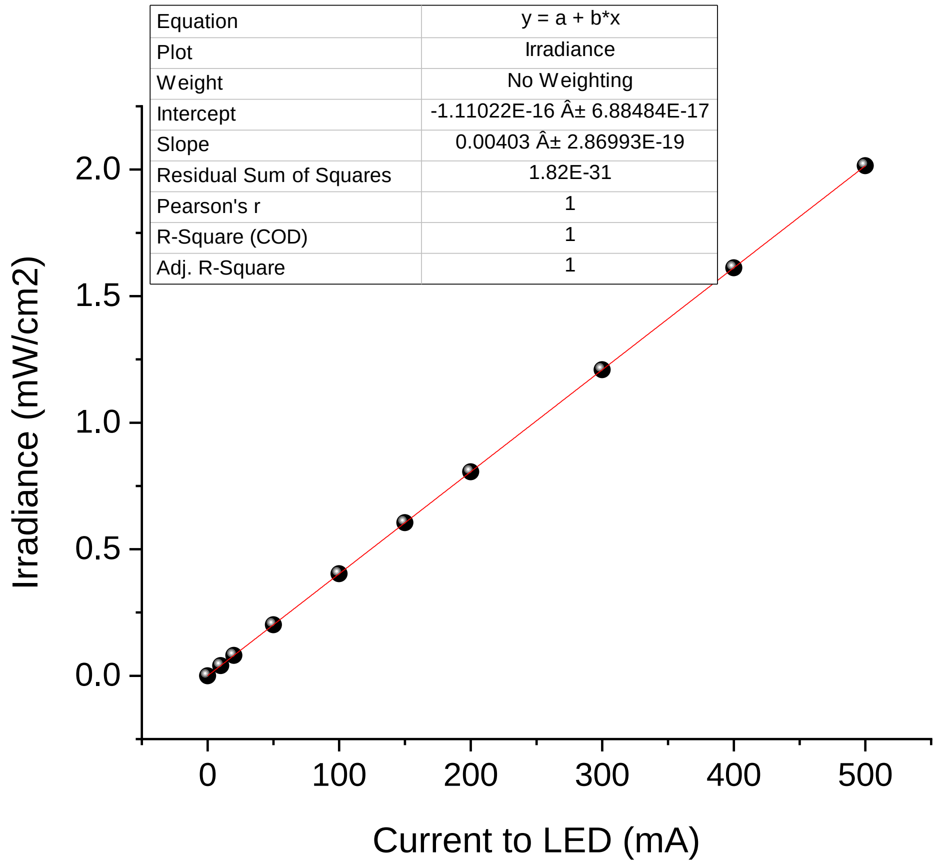

Figure S21. UV light power calibration

We took care to remove the chip from the cantilever holder when performing the calibration. The cantilever partially shadows the UV spot area, underestimating the number of photons arriving to the sample.

14. Bibliography

(1) Berger, R.; Butt, H.-J.; Retschke, M. B. Weber, S. A. L. Electrical Modes in Scanning Probe Microscopy, *Macromol. Rapid Commun.* **2009**, *30*, 1167–1178.

(2) Zhang, Z.; Yates, J. T. Band Bending in Semiconductors: Chemical and Physical Consequences at Surfaces and Interfaces. *Chem. Rev.* **2012**, *112*, 5520−5551.

(3) Y. Huttel. Gas Phase Synthesis of Nanoparticles, Wiley-VCH Verlag GmbH, 2017, ISBN 978-3-527-34060-6.

(4) Martínez, L.; Díaz, M.; Román, E.; Ruano, M.; Llamosa, D. P.; Huttel. Y. Generation of Nanoparticles with Adjustable Size and Controlled Stoichiometry: Recent Advances. *Langmuir* **2012**, *28*, 11241−11249.

(5) Mayoral, A.; Llamosa, D.; Huttel, Y. Novel Co@Au Structure Formed in Bimetallic Core@shell Nanoparticles. *Chemical Communications* **2015,** *51*, 8442-8445.

(6) Martínez, L.; Mayoral, A.; Espiñeira, M.; Roman, E.; Palomares, J.; Huttel, Y. Core@shell, Au@TiO_x Nanoparticles by Gas Phase Synthesis, *Nanoscale*, **2017,** *9*, 6463–6470.

(7) Llamosa, D.; Ruano, M.; Martínez, L.; Mayoral, A.; Roman, E.; García-Hernández, M.; Huttel, Y. The Ultimate Step Towards a Tailored Engineering of Core@Shell and Core@Shell@Shell Nanoparticles. *Nanoscale* **2014**, *6*, 13483-13486.

(8) Díaz, M.; Martínez, L.; Ruano, M. M.; Llamosa, D. P.; Román, E.; García-Hernandez, M.; Ballesteros, C.; Fermento, R.; Cebollada, A.; Armelles, G.; Huttel, Y. Morphological, Structural and Magnetic Characterization of Co Nanoparticles in Silicon Oxide Matrix. *Journal of Nanoparticle Research* **2011**, *13*, 5321–5333.

(9) Ruano, M. M.; Martínez, L.; Huttel, Y. Investigation of the Working Parameters of a Single Magnetron of a Multiple Ion Cluster Source: Determination of the Relative Influence of the

Parameters on the Size and Density of Nanoparticles. *Dataset papers in Nanotechnology* **2013**, Article ID 281672.

(10) R. E. Palmer, R. Cai, J. Vernieres. Synthesis without Solvents: The Cluster (Nanoparticle) Beam Route to Catalysts and Sensors. *Acc. Chem. Res.* 51 (**2018**), 2296-2304.

(11) Ghori, M. Z.; Veziroglu, S.; Hinz, A.; Shurtleff, B. B.; Polonskyi, O.; Strunskus, T.; Adam, J.; Faupel, F.; Aktas, O. C. Role of UV Plasmonics in the Photocatalytic Performance of TiO_2 Decorated with Aluminum Nanoparticles. *ACS Appl. Nano Mater.* **2018**, *1*, 3760-3764.

(12) A. Datta, A.; Porkovich, A. J.; Kumar, P.; Nikoulis, G.; Kioseoglou, J.; Sasaki, T.; Steinhauer, S.; Grammatikopoulos, P.; Sowwan, M. Single Nanoparticle Activities in Ensemble: A Study on Pd Cluster Nanoportals for Electrochemical Oxygen Evolution Reaction. *J. Phys. Chem. C* **2019**, 123, 26124-26135.

(13) Ioannides T.; Verykios, X. E.; Charge Transfer in Metal Catalysts Supported on Doped TiO_2: A Theoretical Approach Based on Metal–Semiconductor Contact *Theory. Journal of Catalysis* **1996**,*161*, 560–569.

(14) Colchero, J.; Gil, A.; Baró, A. M. Resolution enhancement and improved data interpretation in electrostatic force microscopy. *Phys. Rev. B,* **2001**, 64, 245403.

(15) Sacha, G. M.; Rodriguez, F. B.; Serrano, E. and Varona, P. Generalized Image Charge Method to Calculate Electrostatic Magnitudes at the Nanoscale Powered by Artificial Neural Networks. *Journal of Electromagnetic Waves and Applications*, **2010**, 24, 1145-1155.

(16) Bondarenko, A. S.; Ragoisha, G. A. Variable Mott-Schottky Plots Acquisition by Potentiodynamic Electrochemical Impedance Spectroscopy. *J. Solid State Electrochem.* **2005**, *9,* 845–849.

(17) Cardon, F.; Gomes, W. P. On the Determination of the Flat-Band Potential of a Semiconductor in Contact with a Metal or an Electrolyte from the Mott-Schottky Plot. *J. Phys. Appl. Phys.* **1978**, *11*, L63-L67.
